# Building an auxiliary diagnostic and treatment efficacy prediction model for adolescent depression using machine learning based on electroencephalography technology

**DOI:** 10.3389/fnhum.2026.1774822

**Published:** 2026-06-09

**Authors:** Ting Peng, Aiping Chi, Jinghui Yang, Yizhong Ren, Yanru Li, Jing Fan

**Affiliations:** 1 School of Physical Education, Shaanxi Normal University, Xi'an, Shaanxi, China; 2 Shaanxi Vocational and Technical College, Xi'an, Shaanxi, China

**Keywords:** adolescents, depressive symptoms, diagnostic model, electroencephalography, machine learning, predictive model

## Abstract

**Background:**

Adolescent depression, characterized by high incidence rates and significant recurrence risks, has emerged as a major global public health concern. Current diagnosis relies predominantly on subjective questionnaires, lacking objective biomarkers and presenting challenges for early identification. Although exercise intervention is recommended as a safe and effective first-line non-pharmacological treatment, individual responses vary considerably. Approximately 30–40% of patients experience limited symptom improvement, necessitating reliable neurobiological markers to predict intervention response and enable precision exercise prescription. Resting-state electroencephalography (rs-EEG), with its non-invasive nature, low cost, and high temporal resolution, represents an ideal tool for identifying objective markers of depression. This study aims to construct a high-performance auxiliary diagnostic model for adolescent depression based on static and dynamic functional connectivity features of rs-EEG, combined with machine learning algorithms. It further seeks to identify neurophysiological biomarkers capable of predicting exercise intervention efficacy, thereby providing objective and practical technical support for early identification and individualized non-pharmacological interventions.

**Methodology:**

This study employed a machine learning framework combining diagnostic and predictive models. The diagnostic model aimed to identify depressed individuals among healthy adolescents (*N* = 155) and those with subclinical depression (*N* = 149), whilst the predictive model focused on the emotional response to a virtual reality-based exercise intervention within the same depressed cohort (*N* = 149), categorizing participants into high, moderate, and low responders. Six-minute resting-state EEG signals were collected, preprocessed, and segmented into 78 brain regions using the Schaefer template. These regions were then grouped into five core networks (sensory-motor, dorsal attention, salience, default mode, and central executive networks). Forty-five phase-locked functional connectivity features across the theta, alpha, and beta frequency bands were subsequently extracted. A three-stage feature selection method (random forest importance ranking, Pearson correlation redundancy removal, exhaustive search to determine optimal subsets) was employed to screen key neural biomarkers. Four classification models - logistic regression, support vector machines, random forest, and XGBoost - were constructed and compared. Ten-fold cross-validation and grid search were employed to optimize hyperparameters, with performance evaluated using accuracy, recall, precision, F1-score, and AUC-ROC. Finally, the SHAP framework was applied for model interpretability analysis, revealing the contributions and interactions of key brain network features.

**Results:**

Diagnostic model: The XGBoost model demonstrated exceptional performance in distinguishing SCD patients from healthy controls (AUC of 0.994, accuracy of 96.7%). Core biomarkers primarily concentrated on connections within the dorsal attention network (DAN), salience network (SN), and sensorimotor network (SMN) across the *β* and *α* frequency bands, with intra-DAN connections in the *β* band contributing most significantly. SHAP interaction dependency analysis further revealed complex nonlinear interaction patterns that may underlie, including a “fork-collider” synergistic structure centered on *β*-DAN and *θ*-SMN-DAN, cross-frequency inhibition of *β*-DAN by *α*-SN, and dynamic gating of *β*-DMN by *α*-SMN-DAN. The results of this study indicate that subclinical depression (ScD) manifests as a pattern of synergistic dysregulation involving multiple frequency bands and brain networks. In terms of predictive modeling, support vector machines (SVMs) performed best in predicting the response of ScD patients to exercise intervention, with baseline positive affect (PA) levels and functional connectivity between the somatosensory motor network (SMN) and the dorsal attentional network (DAN) in the *β*-band identified as key predictors. Furthermore, the patterns of feature interactions revealed by the model provide a testable framework for understanding the underlying model-derived neurophysiological patterns of subclinical depression from the perspective of multi-frequency, multi-network dyssynchrony; however, these results should still be interpreted as statistical associations rather than direct evidence of biological causality. SHAP analysis revealed an interpretable integration pathway (“behavior-driven → core coordination → dynamic gating → frequency band balance”), providing model-driven insights into the role of behavior-neural integration in personalized interventions.

**Conclusion:**

This study systematically elucidates for the first time the intricate model-derived neurophysiological patterns of subclinical depression at the multi-frequency brain network level. It confirms the substantial potential of EEG functional connectivity as an objective biomarker while providing an interpretable neurophysiological basis for the precise selection of exercise interventions. These findings deepen our understanding of the early pathophysiology of depression and lay a robust foundation for developing low-cost, non-invasive tools for early screening and personalized treatment decision-making.

## 1 Introduction

Depressive symptoms represent a widespread and severe mental disorder, emerging as one of the leading causes of disability and mortality worldwide, imposing a heavy burden on individuals, families, and society. Adolescence, as a critical period for the development of physiological, psychological, and social functioning, is particularly vulnerable to the onset of depression (Pt et al., 2024). Adolescent depression manifests not only as persistent low mood, diminished interest, and anhedonia, but is frequently accompanied by a range of issues including cognitive impairment, declining academic performance, and social withdrawal. More gravely, adolescent depression is closely associated with a marked increase in non-suicidal self-injury (NSSI) behaviors and suicide risk, posing a serious threat to young people’s physical and mental well-being (Leichsenring et al., 2024). Given the high incidence and recurrence rates of adolescent depression, along with its profound negative consequences, achieving early, accurate diagnosis and effective intervention for this condition has become an urgent priority within the field of mental health (Dosenbach et al., 2025; Zheng et al., 2016). However, the current clinical diagnostic framework for adolescent depression faces numerous challenges. The diagnostic process relies primarily on specialist interviews conducted by clinicians and standardized scales based on subjective reports from patients and their families, such as the Hamilton Depression Rating Scale (HAMD) or the Self-Rating Depression Scale (SDS). While these methods are widely employed in clinical practice, their inherent subjectivity limits the objectivity and accuracy of diagnoses. Patients may be unable to provide accurate information due to the stigma associated with mental illness, memory bias, or insufficient self-awareness regarding their emotional state. Furthermore, the symptomatic presentation of adolescent depression exhibits high heterogeneity and frequently overlaps with symptoms of other mental disorders, such as anxiety or attention deficit hyperactivity disorder (ADHD), further complicating differential diagnosis. The time-consuming nature of the diagnostic process may also delay the optimal window for treatment initiation. Consequently, there is an urgent need within both academic and clinical circles to develop objective, quantifiable biological markers to supplement traditional diagnostic methods, thereby enhancing diagnostic precision and efficiency.

In the quest for objective biomarkers of mental disorders, electroencephalography (EEG) has demonstrated considerable potential due to its non-invasive nature, low cost, and millisecond-level temporal resolution. Since its discovery, EEG has served as a vital tool for identifying and investigating numerous neuropsychiatric conditions. Extensive research has confirmed the presence of characteristic abnormal patterns in the resting-state EEG of patients with major depressive disorder (MDD) (Dosenbach et al., 2025). Recent studies concerning adolescent populations have similarly revealed that subclinical and clinically depressed youths exhibit significant differences in resting-state brain electrical activity compared to healthy peers, particularly manifesting as abnormalities in functional connectivity within static networks. These anomalies primarily involve excessive connectivity within the default mode network (DMN), weakened connections between the frontal–parietal control network (FPN) and the default attention network (DAN), and dysregulation of the salience network’s (SN) regulatory functions over other networks (Xu et al., 2024). These findings provide robust neurophysiological evidence for EEG functional connectivity as a potential biomarker for adolescent depression. They lay the theoretical foundation for constructing objective diagnostic and treatment efficacy prediction models based on resting-state EEG network characteristics in this study (Azmi et al., 2022).

Although EEG offers the potential for objective assessment of brain function, the inherent complexity of EEG signals presents significant challenges for data analysis. EEG signals are high-dimensional, non-linear, and non-stationary, with typically low signal-to-noise ratios. Traditional analytical methods, such as visual inspection or simple frequency-domain analysis, often struggle to capture the subtle, variable, and distributed patterns of neural activity associated with complex psychiatric disorders (Cui et al., 2020). Extracting meaningful and reliable features from high-dimensional EEG data that are relevant to the pathophysiological mechanisms of depression remains one of the core challenges facing this field (Ding et al., 2025). This complexity of data urgently requires more advanced and powerful analytical techniques to unlock the rich information contained within EEG signals.

The rise of machine learning (ML) technology offers a powerful solution to this challenge. As a vital branch of artificial intelligence, machine learning excels at automatically learning and recognizing patterns within complex, high-dimensional data, thereby enabling classification or prediction (Azmi et al., 2022). Applying machine learning to EEG analysis effectively overcomes the limitations of traditional statistical methods and manual interpretation, enabling automated, objective, and refined decoding of EEG signals. In recent years, various machine learning algorithms—ranging from classical Support Vector Machines (SVM), K-Nearest Neighbors (KNN), and Random Forests, to more sophisticated deep learning (DL) models such as Convolutional Neural Networks (CNN) and Recurrent Neural Networks (RNN)—have been extensively deployed across diverse domains of EEG signal processing. These include brain-computer interfaces, epilepsy detection, emotion recognition, and psychiatric disorder classification (Sankaran et al., 2022). Particularly with deep learning models, their “end-to-end” learning capability enables direct feature extraction from raw EEG signals, reducing reliance on cumbersome feature engineering dependent on expert knowledge and revolutionizing EEG analysis. The integration of machine learning with EEG technology for auxiliary diagnosis of depression has become a focal point in neuroscience and clinical psychiatric research. Multiple studies have demonstrated that by extracting diverse EEG signal features - such as frequency-domain characteristics, time-domain properties, nonlinear dynamical signatures, and brain network connectivity patterns - and training machine learning classifiers, it is possible to achieve high-precision differentiation between individuals with depression and healthy controls (Lövdal et al., 2021). Recent studies targeting adolescents have also yielded encouraging results. For instance, research employing resting-state EEG signals and affective brain-computer interfaces has demonstrated that a depression detection model, constructed through the integration of multimodal features, achieved an accuracy exceeding 88% on an independent test set. Other studies have focused on EEG microstate analysis, revealing that its time-domain parameters and sequence complexity can effectively distinguish depressed adolescents from healthy controls, achieving classification accuracy rates exceeding 90%. Furthermore, researchers have developed advanced deep learning frameworks (Ding et al., 2025). Such as NSSI-Net, this network is capable of simultaneously capturing both the spatial and temporal dynamics of EEG data, demonstrating exceptional performance in identifying NSSI behaviors highly correlated with depression. Collectively, these studies demonstrate the significant potential of EEG-based and machine learning classification models for the objective, automated-assisted diagnosis of adolescent depression.

Beyond aiding diagnosis, another formidable challenge in clinical practice lies in the individualized selection of treatment regimens. Presently, depression treatment—whether pharmacological or psychotherapeutic—largely continues to follow a trial-and-error approach. Patient responses to identical treatment protocols vary considerably, with approximately one-third showing no response to initial therapy (Gan et al., 2021). This uncertainty not only prolongs patients’ suffering but also leads to a waste of healthcare resources. Consequently, identifying biomarkers capable of predicting treatment response to achieve “precision psychiatry” represents a crucial direction for advancement in this field. As a direct reflection of brain function, the baseline state of EEG prior to treatment may harbor key information for predicting subsequent therapeutic efficacy (Qi et al., 2025). This uncertainty not only prolongs patients’ suffering but also leads to a waste of healthcare resources. Consequently, identifying biomarkers capable of predicting treatment response to achieve “precision psychiatry” represents a crucial direction for advancement in this field. As a direct reflection of brain function, the baseline state of EEG prior to treatment may harbor key information for predicting subsequent therapeutic efficacy. Existing research has begun to explore the association between baseline neurophysiological indicators and treatment outcomes. For instance, in repetitive transcranial magnetic stimulation (rTMS) therapy, pre-treatment EEG frequency-specific power ratios and event-related potential (ERP) components have been found to correlate with treatment efficacy. Applying machine learning models to this field holds promise for constructing predictive models capable of forecasting individual patient responses to specific therapies based on pre-treatment EEG characteristics. This approach could provide clinicians with a scientific basis for developing personalized treatment plans.

Despite significant advances in existing research, several pressing issues remain in applying EEG- and machine learning-based models to the clinical practice of adolescent depression. Firstly, the majority of studies have focused on adult populations, with relatively fewer investigations targeting adolescents as a distinct cohort. Given that the adolescent brain continues to undergo substantial development and restructuring, the neurophysiological mechanisms of depression in this age group may differ from those in adults. Consequently, directly applying adult models to adolescents may prove inappropriate. Secondly, existing research predominantly focuses on “diagnostic” tasks - distinguishing patients from healthy individuals - while neglecting equally clinically significant “predictive” tasks, such as forecasting treatment efficacy. Studies integrating diagnosis and efficacy prediction within a unified analytical framework remain exceedingly rare. Finally, many high-performance machine learning models, particularly deep learning models, are frequently criticized as “black boxes” due to the lack of transparency and interpretability in their decision-making processes. To gain clinicians’ trust and adoption of these models, it is crucial to develop not only accurate but also interpretable models. This facilitates the identification of key biomarkers underpinning model decisions, thereby deepening our understanding of the model-derived neurophysiological patterns (All interpretations are model-based and correlational) underlying the disorder. In light of this, the present study aims to address the aforementioned research gap by developing a comprehensive framework based on resting-state EEG data and machine learning techniques. This framework seeks to resolve two core clinical challenges: the auxiliary diagnosis of adolescent depression and the prediction of treatment efficacy. The specific objectives of this study include: (1) Acquiring resting-state EEG data from adolescent patients with depression and healthy controls to construct a high-precision machine learning model for the objective auxiliary diagnosis of adolescent depression; (2) Developing a machine learning model capable of predicting treatment outcomes using pre-treatment baseline EEG data within a cohort of patients receiving standardized treatment protocols; (3) Employing advanced feature selection and model interpretation techniques to identify key EEG biomarkers in diagnostic and predictive models, thereby providing novel neuroscientific insights into the pathophysiological mechanisms of adolescent depression and its response to treatment; (4) Systematically evaluating the performance, robustness, and generalizability of the constructed models, laying the groundwork for their future translational application as clinical decision support tools. Through this research, we aim to advance the transition of mental health diagnosis and treatment from subjective experience to an objective, data-driven precision medicine paradigm, ultimately improving clinical outcomes and quality of life for adolescents with depression. This study selected the sensorimotor network (SMN), dorsal attentional network (DAN), salience network (SN), default mode network (DMN), and central executive network (CEN) as the core analytical framework, primarily based on the following neurobiological evidence: multimodal imaging studies have demonstrated significant functional dysregulation in these five networks in adolescents with depression. Among these, the attention-salience imbalance in the DAN and SN, excessive introspection in the DMN, and abnormal mind–body interaction in the SMN have demonstrated potential as early biomarkers in the subclinical stage. Based on this, we propose two hypotheses: H1: Functional connectivity across the five networks in the theta, alpha, and beta frequency bands will exhibit significant multi-frequency, multi-network dysregulation patterns in adolescents with subclinical depression; Baseline network connectivity characteristics can effectively predict the degree of positive emotional improvement following VR-combined exercise intervention. Although this study employs data-driven machine learning as the primary analytical method, the aforementioned hypotheses provide a clear neurobiological orientation for feature extraction and model construction.

## 2 Materials and methods

### 2.1 Experimental subjects

This study comprises two research designs: diagnostic models and predictive models. The diagnostic model sample consisted of adolescents aged 13 to 18 years, including a healthy group (*N* = 155) and a depression group (*N* = 149). Depressive symptoms were assessed using the Centre for Epidemiologic Studies Depression Scale and the Patient Health Questionnaire-9, with screening criteria set at CES-D scores ≥20 and PHQ-9 scores ≥10, respectively. All assessment results were verified by professional counsellors from the school’s psychological counselling centre. The predictive model focused on forecasting the positive affect improvement effects of virtual reality combined with exercise intervention on adolescents with depressive symptoms (*N* = 149). All participants met the following inclusion criteria: no history of alcohol abuse or substance misuse, no known physical illnesses or developmental abnormalities, and signed informed consent. This study protocol received ethical review approval from the Academic Committee of Shaanxi Normal University (Approval No.: 202516012). Written informed consent was obtained from all participants and their legal guardians prior to participation. All procedures were conducted in accordance with the Declaration of Helsinki.

### 2.2 EEG signal acquisition and preprocessing

**Figure 1:**
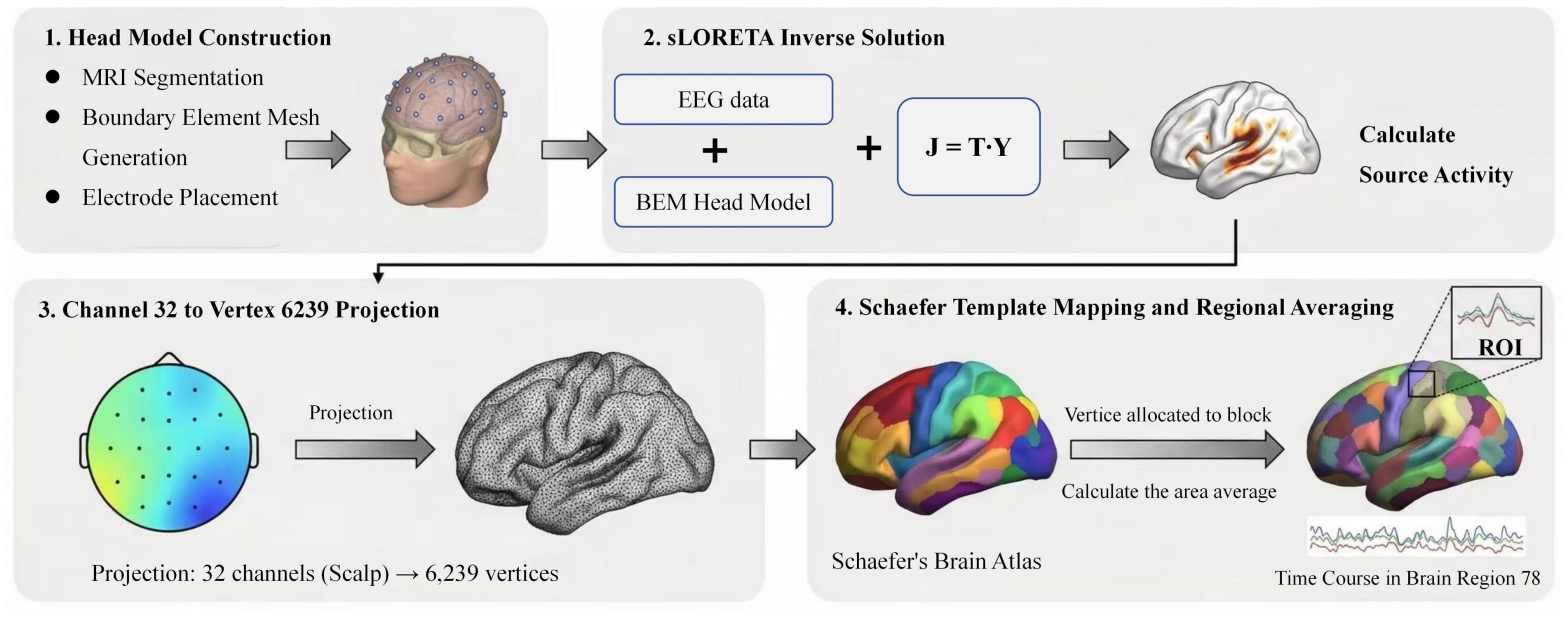
Schematic diagram of the source reconstruction and Schaefer template mapping process. All scalp channels are projected into cortical source space using the BEM head model and then mapped to the Schaefer-78 blocks.

This experiment was conducted in a dimly lit, quiet, soundproofed room to minimize external environmental interference during electroencephalographic signal acquisition. As illustrated in the flowchart (Figure 1), subjects underwent scalp cleansing prior to the experiment to optimize electrode-scalp contact quality. EEG signals were acquired using a 32-channel EEG recording system (Brain Vision Recorder, Neuroscan, United States) integrated with Curry 8.0 software. Six minutes of resting-state EEG data were recorded from both the student group with depressive symptoms (ScD) and the healthy control group (HC). Electrode-to-scalp contact impedance was strictly maintained below 10 kΩ, with a sampling frequency set at 1024 Hz. Throughout the experiment, researchers verbally instructed participants to minimize blinking and head movement to reduce interference from eye movements and myoelectric artifacts, thereby ensuring data quality.

Data preprocessing was performed using the EEGLAB 2013 toolbox in the MATLAB 2022b environment. First, irrelevant channels (VEOG/HEOG) were removed. Subsequently, a 48–52 Hz notch filter was applied to completely eliminate 50 Hz power line noise, followed by a 1–30 Hz bandpass filter to retain signals of interest in the theta, alpha, and beta bands while removing ultra-low-frequency drift and ultra-high-frequency components. This sequence ensures that power line noise is completely filtered out before entering the bandpass filter, thereby avoiding redundant filtering. Meanwhile, broad-spectrum high-frequency interferences such as muscle artifacts and eye movements are primarily identified and removed through subsequent Independent Component Analysis (ICA), rather than relying on filters. Any data points in any channel with an absolute amplitude exceeding ±70 μV were considered outliers and completely excluded; no interpolation was performed to ensure data integrity. After this step, the data retention rate remained as high as 96.2%, and there was no significant impact on subsequent network analysis. Additionally, only EEG data with a duration exceeding 300 s were retained for subsequent analysis to ensure data accuracy and reliability.

Using EEGLAB’s runica algorithm for independent component analysis (ICA), artifacts such as eye movements and muscle activity were separated and removed. After artifact removal (primarily targeting eye-movement, muscle-related, and cardiac components identified using ICALabel and manual review), an average of 25.4 ± 2.1 independent components were retained for each subject, accounting for 84.7% of the original components.

### 2.3 Network partitioning and feature extraction

Based on the Schaefer template, we performed a detailed segmentation of the cerebral cortex and identified 78 brain regions. This template integrates the functional characteristics and anatomical structures of brain regions, providing high-precision regional localization for studies on functional connectivity in brain networks. Based on the similarity between functional connectivity and cognitive functions, these 78 regions were integrated into five core networks: the sensorimotor network (SMN), the dorsal attentional network (DAN), the salience network (SN), the default mode network (DMN), and the central executive network (CEN). This integration balances regional specificity with network integrity, facilitating systematic analysis of interactions among networks and their roles in cognitive and emotional processing. To quantify the strength of functional connectivity between networks, this study employed phase lock value (PLV) as the primary metric, with all functional connectivity metrics calculated in source space. The specific procedure is as follows: Preprocessed EEG signals were source-reconstructed using the FieldTrip toolbox (v2023.01, MATLAB 2022b). The standardized low-resolution electromagnetic tomography (sLORETA) algorithm was employed, based on an individual boundary element method (BEM) head model and MNI standard space registration, to back-project the signals onto 6,239 cortical vertices; Subsequently, brain regions are averaged according to the Schaefer-78 template, the instantaneous phase of each brain region is extracted (see Figure 1), and PLV is calculated. To further reduce common-reference effects, average reference re-referencing is employed throughout the process, and a surface Laplace transform is applied prior to source reconstruction. Multiple anti-leakage strategies have effectively reduced the impact of volumetric conduction, resulting in higher biological reliability compared to direct calculations in sensor space.

PLV values range from [0,1]; values closer to 1 indicate higher phase synchrony and stronger functional connectivity between brain regions, while values closer to 0 indicate lower synchrony and weaker functional connectivity. Its mathematical expression is:

$$\mathrm{PLV}=\frac{1}{T}|\sum_{t=1}^{T}e^{i(\phi_{i}(t)-\phi_{j}(t))}|$$

Here, T denotes the total number of time points within the time window, while $\phi_{i}(t)$ and $\phi_{i}(t)$ represent the instantaneous phase of brain regions *i* and *j* at time t, respectively. By calculating the PLV values between networks, we obtain quantitative metrics for functional connectivity strength, providing a reliable data foundation for analyzing inter-network interaction patterns and their dynamic changes before and after intervention. The optimized algorithm ensures computational efficiency and result accuracy, enhancing the scientific rigor and interpretability of detailed static network analysis. By calculating PLV values across five networks and their interactions within three frequency bands, a total of 45 functional connectivity features were extracted. This provides a robust data foundation for analyzing dynamic interaction patterns between networks and their changes before and after intervention. The optimized algorithm improves computational efficiency and result precision, thereby strengthening the scientific validity and interpretability of static network analysis.

### 2.4 Intervention programs and effectiveness evaluation

This study employed a virtual reality (VR)-enhanced aerobic exercise intervention program, comprising VR-guided moderate-intensity cycling and group gamified exercise (30 min per session, three times weekly for 8 weeks, maintaining heart rate at 60–75% of maximum heart rate), supervised by professional trainers. Following the intervention, participants were reassessed using the Positive Affect (PA) subscale of the Positive and Negative Affect Schedule (PANAS). To ensure clinical relevance in subsequent analyses, the study employed Jacobson and Truax’s (1991) clinical significance methodology, utilizing a dual criterion of the Reliable Change Index (RCI) and clinical cutoff point (C) to refine the classification of intervention outcomes.

Clinical Cut-off Point (C) Calculation:

$$C=\frac{SD_{\mathrm{HC}}\times M_{\mathrm{SCD}}+SD_{\mathrm{SCD}}\times M_{\mathrm{HC}}}{SD_{\mathrm{HC}}+SD_{\mathrm{SCD}.}}$$

Based on the PA norm for healthy individuals (30.083 ± 1.867) and the patients’ baseline data (19.26 ± 2.813), the clinical cut-off point *C* = 26 points was calculated. This score represents the functional boundary distinguishing the “patient group” from the “healthy group”. For calculating the Reliable Change Index (RCI) threshold, the standard deviation of the difference is first computed using the following formula:

$$S_{\text{diff}}=SD_{\text{baseline}}\times \sqrt{2\times (1-r_{\mathrm{xx})}}$$

Among these, the baseline standard deviation $SD_{\text{baseline}}$ = 2.813, and the reliability coefficient $r_{\mathrm{xx}}$ for the PANAS scale is 0.86.

$$\mathrm{RC}I_{\text{critical}}=1.96\times S_{\text{diff}}$$

The figure 1.96 represents the confidence coefficient (corresponding to a significance level of *p* < 0.05).

In summary, the final calculation yields an RCI threshold of 3 points. This value indicates that improvement scores ≥3 points exceed measurement error and possess statistical significance. Based on the aforementioned criteria, the RCI threshold categorizes patients’ post-intervention responses into three groups: (1) High responders (recovery/significant improvement): Improvement score ≥3 points and post-intervention PA ≥ 26 points. These patients not only demonstrate marked improvement but also achieve functional recovery to healthy levels. (2) Moderate responders (improvement without full recovery): Improvement score ≥3 points but PA < 26 points post-intervention. These patients show significant improvement but do not fully regain functional capacity within the healthy range. (3) Low responders (limited improvement): Improvement score <3 points. The improvement in these patients does not reach a statistically significant level.

### 2.5 Model construction and interpretability analysis

This study utilized functional connectivity metrics derived from electroencephalograms to sequentially construct two machine learning models with distinct clinical objectives: one for diagnosing depressive symptoms, and another for predicting intervention outcomes within patient cohorts. To mitigate the risk of data leakage commonly encountered in machine learning applications, this study establishes a strict “data isolation” principle within its overall analytical framework: all data is pre-divided into strictly independent training and testing sets before undergoing feature engineering and model training; simultaneously, a pipeline mechanism is employed within the cross-validation process to perform data standardization, ensuring the objectivity of model performance evaluation and the model’s true generalization capability.

#### 2.5.1 Diagnostic model

The purpose of this model is to accurately identify individuals exhibiting depressive symptoms within a mixed population comprising both healthy and depressed individuals.

The initial full dataset for this study comprised a total of 304 participants (including *N* = 155 in the healthy control group and *N* = 149 in the subclinical depression group). Prior to formally initiating the modeling process, to prevent any leakage of test set information into the training phase from the outset, this study employed a stratified sampling strategy, pre-dividing these 304 complete independent samples into a training set (*N* = 243) and an independent test set (*N* = 61) in an 8:2 ratio. This test set was strictly sealed off and remained completely invisible to the subsequent feature selection and model training processes. The initial feature set consisted of 45 phase-locked value metrics calculated based on five core brain networks across three frequency bands (*θ*, *α*, *β*). To construct a model with high discriminatory performance, this study implemented a series of model optimization measures. First, to extract key neural biomarkers from high-dimensional features, we combined random forests, Pearson correlation coefficients, and exhaustive search to form a systematic three-stage feature selection process, aiming to construct an optimal feature subset and reduce the adverse effects of feature redundancy on the model. Second, we developed and compared four classification models: logistic regression, support vector machines (SVMs), random forests, and XGBoost. All models were trained using 10-fold cross-validation, and hyperparameters were optimized via grid search. To prevent “local leakage” during this process, we implemented a pipeline mechanism. This mechanism strictly ensures that data standardization (e.g., *Z*-score transformation) in each cross-validation iteration calculates the mean and standard deviation based solely on the distribution characteristics of the current 9-fold training data, and subsequently applies these parameters to the remaining 1-fold validation data, thereby completely severing the pathway through which the distribution information of the validation set could be “peeked at” in advance by the training set. Finally, to objectively evaluate and compare the classification performance of each model and select the model with the best identification performance, this study adopted accuracy, recall (sensitivity), precision, F1-score, and the area under the receiver operating characteristic curve (AUC-ROC) as core evaluation metrics. Among these, AUC-ROC is used as the primary metric to assess the model’s overall discriminatory ability, while the F1-score is used to comprehensively evaluate the model’s performance on the minority class (the depression group).

#### 2.5.2 Predictive models

The predictive model aims to forecast individual responses to improvements in positive emotions among adolescents with subclinical depression following a VR-combined aerobic exercise intervention. The model utilized a sample of 149 adolescents with subclinical depression who underwent a VR-guided moderate-intensity aerobic exercise intervention. Subsequently, participants were classified into high-response, moderate-response, and low-response groups (preset as a three-class target variable) based on changes in positive affect (PA) before and after the intervention, using the Jacobson and Truax clinical significance criteria (RCI and clinical cutoff values). The input features for this model directly adopt the optimal subset of six features screened by the diagnostic model, supplemented by baseline PA scores as an additional clinical indicator. This design is primarily based on the hypothesis that “the baseline neural network state of depression constitutes the biological basis for intervention response.” This approach not only helps maintain consistency in the feature space between diagnostic and predictive models—facilitating comparisons of how the same neurobiological marker functions differently in diagnostic versus prognostic tasks—but also organically integrates stable brain network biomarkers with key clinical status indicators. Although diagnostic and prognostic mechanisms may differ, this study prioritized feature transfer strategies to ensure model interpretability and cross-task continuity. The study employed the same four machine learning algorithms as the diagnostic model (SVM, Random Forest, Logistic Regression, XGBoost), all of which utilized 10-fold cross-validation combined with grid search for hyperparameter optimization. The construction of this predictive model strictly adheres to the aforementioned data leakage prevention mechanism: first, a test set is set aside independently in an 8:2 split, and feature standardization is dynamically performed internally via a pipeline mechanism during 10-fold cross-validation to ensure the reliability of the treatment efficacy predictions. In model evaluation, in addition to conventional metrics such as accuracy, precision, recall, F1-score, and AUC-ROC, particular emphasis was placed on the ability to identify “high responders”; therefore, recall and F1-score were used as core evaluation metrics, while AUC-ROC was used to assess overall performance.

#### 2.5.3 Key feature selection

In brain imaging studies, a vast array of brain network topological metrics derived from neurophysiological activity - such as the clustering coefficient, characteristic path length, and node centrality - exhibit complex collinearity relationships. To precisely identify key biomarkers most relevant to clinical states (such as disease diagnosis and cognitive scores) from these high-dimensional features, this study employs a systematic, three-stage feature selection method. To prevent selection bias from causing information from the test set to be incorporated into the model prematurely (i.e., feature selection leakage), the entire three-stage feature selection process in this study was strictly confined to the independent 80% training set and executed independently within it. The three-stage feature selection method used in the diagnostic model is as follows:

1. First, a random forest model was employed to assign importance scores to all 45 features, identifying the top 15 most significant ones. The random forest quantifies each feature’s contribution to classification by evaluating the mean reduction in impurity at decision nodes. This method effectively captures the complex nonlinear relationships between brain network features and clinical phenotypes, generating a feature sequence ranked in descending order of importance.
2. Subsequently, a forward selection strategy was applied to the 15 identified key features, using a Pearson correlation coefficient threshold (|*ρ*| = 0.85) to sequentially eliminate secondary features highly correlated with those of high importance. This process ensured retention of the most discriminative neurobiological information while mitigating model instability caused by multicollinearity.
3. Finally, an optimal subset was determined through exhaustive forward search. This method systematically evaluated the generalization performance of feature subsets of varying sizes (S₁ ⊂ S₂ ⊂ . ⊂ S_K_) via five-fold cross-validation, plotting performance-versus-feature-count curves. The minimal feature subset corresponding to peak performance was selected, achieving an optimal balance between model complexity and predictive capability.

#### 2.5.4 Model interpretability analysis

To gain a deeper understanding of the decision-making mechanisms of the best model and extract neuroscientific insights, while ensuring that the extracted biomarkers possess genuine generalizability and avoiding explanatory overfitting to the training data (Explanation Bias), the SHAP framework used in this study for visual interpretation is strictly based on the independent test set that was previously set aside. The following are several methods for conducting explanatory analysis of the model:

1. Global Explanation: First, SHAP summary plots were utilized to display the average impact magnitude and distribution of all features on model outputs, thereby globally identifying the most critical brain network features distinguishing healthy from depressed groups.
2. Feature Association Analysis: We constructed a joint visualization of feature importance versus label relevance, comparing SHAP feature importance with their correlation to the target category (healthy/depressed). This analysis calculates the Pearson correlation coefficient between each feature and the target category, then compares this with SHAP importance rankings. This aims to discern whether a feature’s contribution to model prediction stems from its independent discriminative power within the complex model, or primarily from a simple linear association with the target variable. This comparison helps reveal whether the model has learned deep feature interaction patterns beyond traditional linear relationships.
3. Building upon this, main effect plots quantify the independent contribution of individual features; interaction heatmaps systematically reveal synergistic or antagonistic effects between different brain network features; and dependency plots provide an in-depth illustration of how variations in key feature values and their interactions collectively influence the model’s final decision.

The aforementioned analyses not only technically validate the model’s decision-making logic but also aim to elucidate the brain network dysregulation mechanisms underlying depressive symptoms from a neuroscientific perspective. Furthermore, they preliminarily explore predictive biomarkers associated with intervention response.

### 2.6 Mathematical statistics and analysis

All statistical analyses and visualizations were conducted in a Python 3.9 environment, primarily utilizing the following libraries: scikit-learn 1.2.2, SciPy 1.10.1, statsmodels 0.13.5, Pandas 1.5.3, Matplotlib 3.7.1, and Seaborn 0.12.2. Unless otherwise specified, statistical significance was set at *p* < 0.05 (two-tailed test). Effect sizes were reported using measures including Cohen’s d (t-tests), η^2^ (analysis of variance), and Cramér’s V (chi-square tests) to provide a quantitative assessment of clinical relevance.

## 3 Results

### 3.1 Diagnostic model sample basic information and predictive model sample basic information

This study included 149 adolescents with subclinical depression (ScD group) and 155 healthy control adolescents (HC group) for diagnostic model construction. The demographic and clinical characteristics of both groups are presented in Table 1. Chi-square tests revealed no significant differences between the groups in gender, handedness, Tanner stage of puberty, or parental educational attainment (*p* > 0.05). Independent samples *t*-tests revealed that the ScD group exhibited a significantly lower annual household income than the HC group (11.2 ± 3.8 vs. 12.5 ± 4.2, t = −2.89, *p* = 0.004). As anticipated, the ScD group exhibited significantly higher CES-D scores (22.5 ± 5.1 vs. 8.3 ± 3.2, t = 28.45, *p* < 0.001) and PHQ-9 scores (10.2 ± 2.5 vs. 2.1 ± 1.8, *t* = 31.67, *p* < 0.001) compared to the HC group. No statistically significant differences were observed between the groups for other variables (age, BMI, etc.) (*p* > 0.05). In summary, apart from the anticipated group differences in household economic status and severity of depressive symptoms, the two groups were highly matched on other key demographic variables, demonstrating good comparability.

**Table 1:** Comparison of demographic and basic characteristics between adolescents with depressive symptoms and healthy control adolescents.

Category	Indicator	ScD	HC	Statistical test value		
		*N* = 149	*N* = 155	χ^2^	*t*	*p*-value
	Age	15.8 ± 1.4	15.5 ± 1.6		1.750	0.081
	Annual household income	11.2 ± 3.8	12.5 ± 4.2		−2.89	0.004
Demographics	Gender (male/female, %)	64 (43.0%)/ 85 (57.0%)	80 (51.6%)/ 75 (48.3%)	2.560		0.110
	Dominant hand (left/right/both, %)	Left (6.7%)/ Right (88.6%)/ Mixed (4.7%)	Left (5.3%)/ Right (91.3%)/ Mixed (3.3%)	0.82		0.664
	Parental Educational Attainment (High School and Above, %)	Father (83.9%)	Father (88.0%)	1.030		0.310
		Mother (79.2%)	Mother (84.0%)	1.150		0.284
	Adolescent developmental stage (tanner stage, %)	Phase I (0.0%)	Phase I (0.0%)	0.840		0.932
		Phase I (8.1%)	Phase I (10.0%)			
		Phase III (30.2%)	Phase III (33.3%)			
		Phase IV (48.3%)	Phase IV (45.3%)			
		Phase V (13.4%)	Phase V 11.3%)			
Scale score	CES-D	22.5 ± 5.1	8.3 ± 3.2		*t* = 28.45	*p* < 0.001
	Phq-9	10.2 ± 2.5	2.1 ± 1.8		*t* = 31.67	*p* < 0.001

This study included 149 patients with sickle cell anemia who completed a 12-week exercise intervention. Participants were divided into two groups based on the degree of improvement in positive affect (see Table 2): the high-response group (ΔPA ≥ 3 points and post-intervention PA ≥ 26 points, *N* = 87, 58.4%) and the moderate-response group (ΔPA ≥ 3 points and post-intervention PA < 26 points, *N* = 62, 41.6%). The low-response group (ΔPA < 3 points) was completely absent, highlighting the good efficacy of the current VR-enhanced exercise program in this subclinical cohort rather than insufficiently strict grouping criteria; therefore, this group was excluded from the final analysis (see Table 2). Baseline comparisons showed that the PA score in the high-response group was significantly higher than that in the moderate-response group (20.11 ± 1.07 vs. 18.79 ± 1.14, *t* = 7.29, *p* < 0.001). Following the intervention, PA levels in both groups improved compared to baseline. However, the endpoint PA score in the high-response group (27.57 ± 1.52) was significantly higher than that in the moderate-response group (24.21 ± 1.06; *t* = 15.23, *p* < 0.001), and the high-responder group also exhibited a greater magnitude of improvement in positive affect (PA) (∆PA) (7.46 ± 1.37 vs. 5.42 ± 1.06, *t* = 10.18, *p* < 0.001). These findings suggest that high responders have higher baseline levels of positive affect and demonstrate greater absolute benefits and relative response gains following exercise intervention. This provides key clinical stratification criteria for the subsequent development of precision efficacy prediction models.

**Table 2:** Distribution of exercise-intervention response groups and changes in positive affect scores.

Indicator	High-response group *N* = 87 (58%)	Moderate-response group *N* = 62 (42%)	Low-response group *N* = 0 (0%)	*t*	*p*
Baseline PA	20.11 ± 1.23	18.79 ± 1.14	--	7.29	*p* < 0.001
Post-intervention PA	27.57 ± 1.52	24.21 ± 1.06	--	15.23	*p* < 0.001
PA improvement score (ΔPA)	7.43 ± 1.37	5.43 ± 1.06	--	10.18	*p* < 0.001

### 3.2 Results of optimal feature subset selection for diagnostic models

This study employed the Schaefer template to partition the cerebral cortex into 78 brain regions, which were then integrated into five core networks based on functional and cognitive characteristics: the sensorimotor network (SMN), the dorsal attention network (DAN), the salience network (SN), the default mode network (DMN), and the central executive network (CEN). Phase-locked value (PLV) was used as the functional connectivity metric. Connectivity strength was calculated within and between the five networks across the theta, alpha, and beta frequency bands, yielding 45 features for constructing a diagnostic model for depressive symptoms. To enhance model generalization, mitigate overfitting risks, and reduce computational complexity, a multi-stage feature selection framework was applied to systematically optimize the initial 45 features.

**Figure 2:**
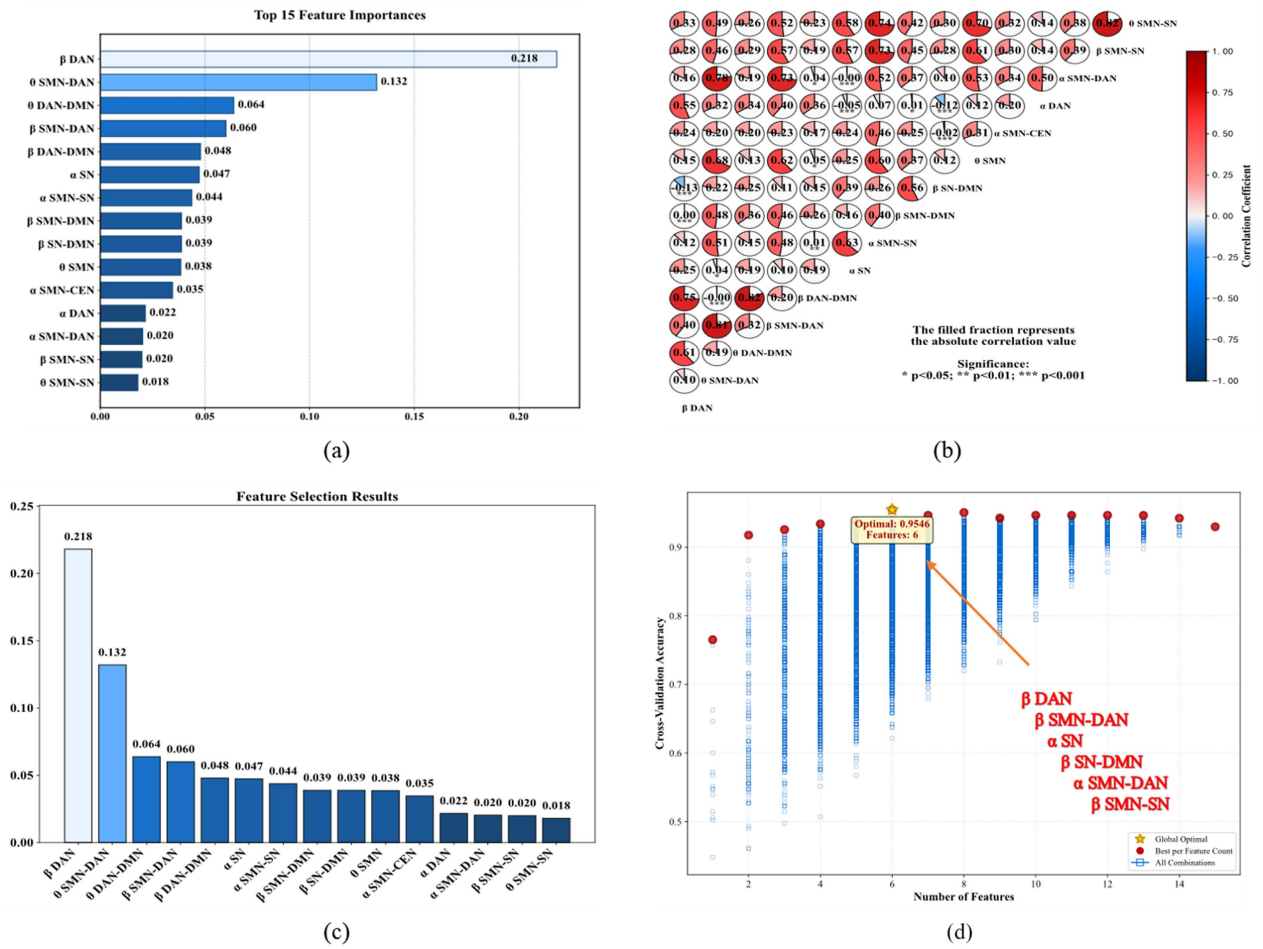
Feature selection procedure for the diagnostic model. This figure illustrates the multi-step feature selection procedure used to identify the optimal EEG functional connectivity biomarkers for the diagnostic model. **(a)** Random forest-based importance ranking of the top 15 candidate features among the initial 45 PLV-based functional connectivity features. **(b)** Pearson correlation matrix of the top 15 candidate features. The color scale represents the direction and magnitude of pairwise correlations, and significance levels are indicated where applicable. **(c)** Features retained after collinearity screening. This panel lists the candidate features that remained after applying the Pearson correlation threshold and shows their corresponding importance values. **(d)** Exhaustive subset search for determining the optimal diagnostic feature subset. Model performance was compared across feature subsets, and the final six-feature subset was selected based on the best balance between model performance and feature parsimony.

The flowchart for biomarker screening is shown in Figure 2. The top 15 biomarkers ranked by feature importance, calculated using a random forest classifier, are shown in Figure 2a, with the *β*-band DAN, *θ*-band SMN-DAN, *θ*-band DAN-DMN, and *β*-band SMN-DAN occupying the foremost positions. Figure 2b presents a Pearson correlation heatmap among these 15 biomarkers, where the color intensity denotes correlation strength (red indicates positive correlation, blue negative). Each cell’s pie chart fill proportion corresponds to the absolute value of the correlation coefficient, annotated with asterisks for significance levels (*p < 0.05, **p < 0.01, ***p < 0.001). Figure 2c details the specific names and importance scores of the features retained after collinearity filtering. Overall, the correlations among the top 15 features in this study were low, and no high collinearity (|*r*| > 0.85) was observed. Consequently, all 15 high-importance features were retained for subsequent modeling analyses, indicating that each feature contributes relatively independently to the diagnostic model. Figure 2d employs a forward exhaustive search method to evaluate the performance of different subsets of retained features after redundancy removal. This process ultimately identified six feature subsets that optimise model diagnostic performance, ranked as follows: *β*-band DAN, *β*-band SMN-DAN, *α*-band SN, *β*-band SN-DMN, *α*-band SMN-DAN, and *β*-band SMN-SN.

### 3.3 Diagnostic models and diagnostic model construction results

#### 3.3.1 Diagnostic model

In this study, we systematically evaluated the classification performance of four machine learning algorithms: XGBoost, Random Forest (RF), Support Vector Machine (SVM), and Logistic Regression (LR) to construct a diagnostic model for depressive symptoms. These models were trained and tested on optimised subsets of six features (*β*-band DAN, *β*-band SMN-DAN, *α*-band SN, *β*-band SN-DMN, *α*-band SMN-DAN, and *β*-band SMN-SN), with the training and testing datasets divided in an 8:2 ratio. The samples from the HC group were labelled as 0, while the samples from the SCD group were labelled as 1. To ensure robustness, all models underwent performance evaluation via 10-fold cross-validation. The evaluation metrics included accuracy, precision, recall, F1 score, and area under the ROC curve (AUC), where higher AUC values indicate superior overall classification performance.

**Figure 3:**
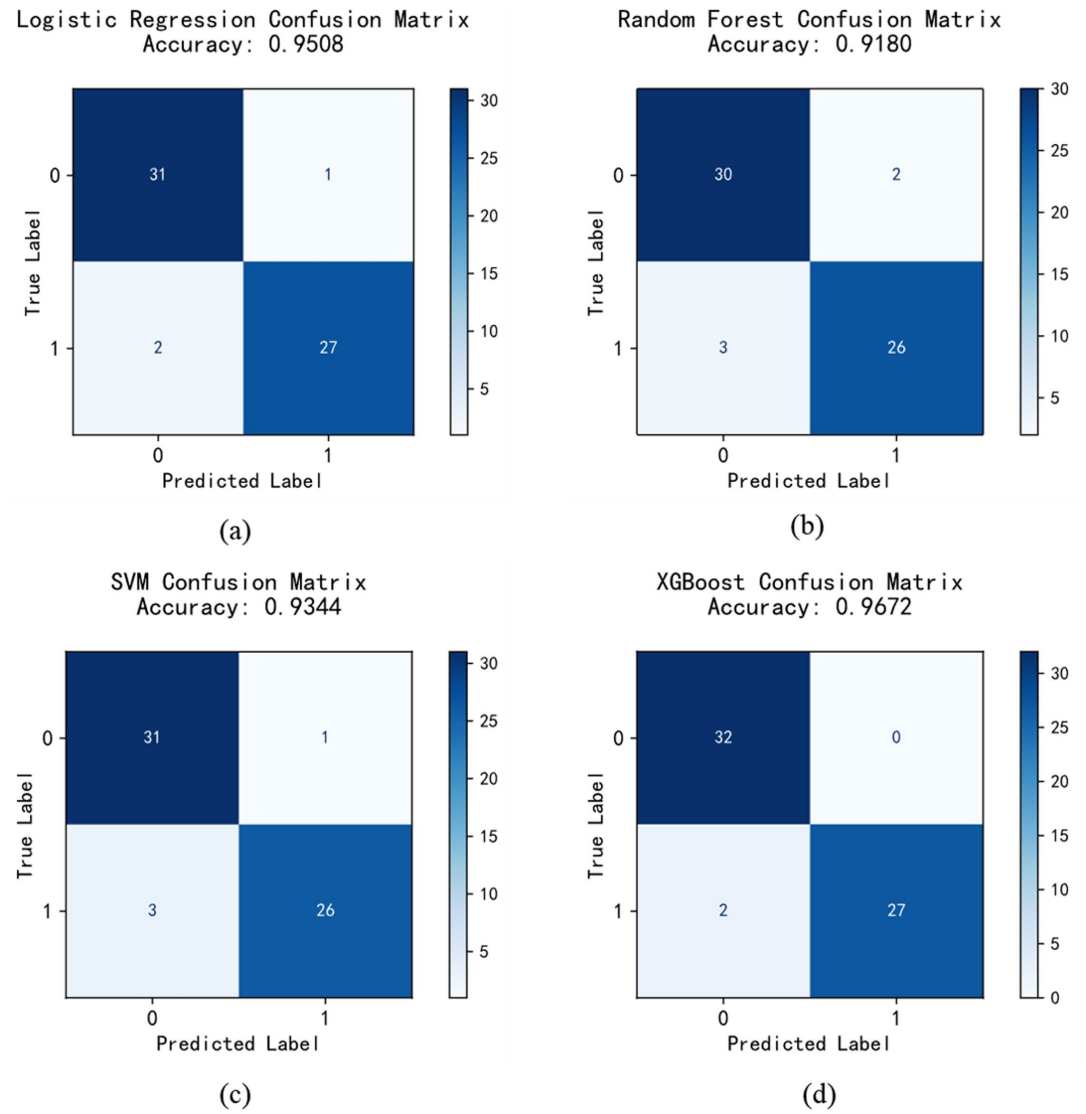
Confusion matrix for different machine learning models in diagnostic tasks. This figure presents the confusion matrix results for four mainstream machine learning models on the validation set: **(a)** Logistic Regression; **(b)** Random Forest; **(c)** Support Vector Machine; and **(d)** XGBoost. The horizontal axis denotes the predicted category (0: healthy control group, 1: subclinical depression group), while the vertical axis represents the true category. Diagonal values indicate correctly classified samples, and off-diagonal values denote misclassified samples. The color intensity reflects the sample quantity, with darker shades indicating larger sample sizes.

The confusion matrix analysis (Figure 3) indicates that the XGBoost model demonstrated optimal performance in the binary classification task between depressive symptoms (ScD) and healthy controls (HC). It achieved the highest true positive rate (TP) and true negative rate (TN), alongside the lowest false negative rate (FN). This suggests that the model possesses significant advantages in both identifying patients with depressive symptoms (sensitivity) and excluding healthy individuals (specificity). In contrast, while the Random Forest model demonstrated superior recall, its elevated false positive rate (FP) resulted in reduced precision. The Support Vector Machine model achieved a moderate balance between precision and recall, yet its overall misclassification rate remained higher than that of XGBoost. As a linear baseline model, Logistic Regression exhibited a notably high false negative rate, revealing insufficient sensitivity in detecting positive cases.

**Figure 4:**
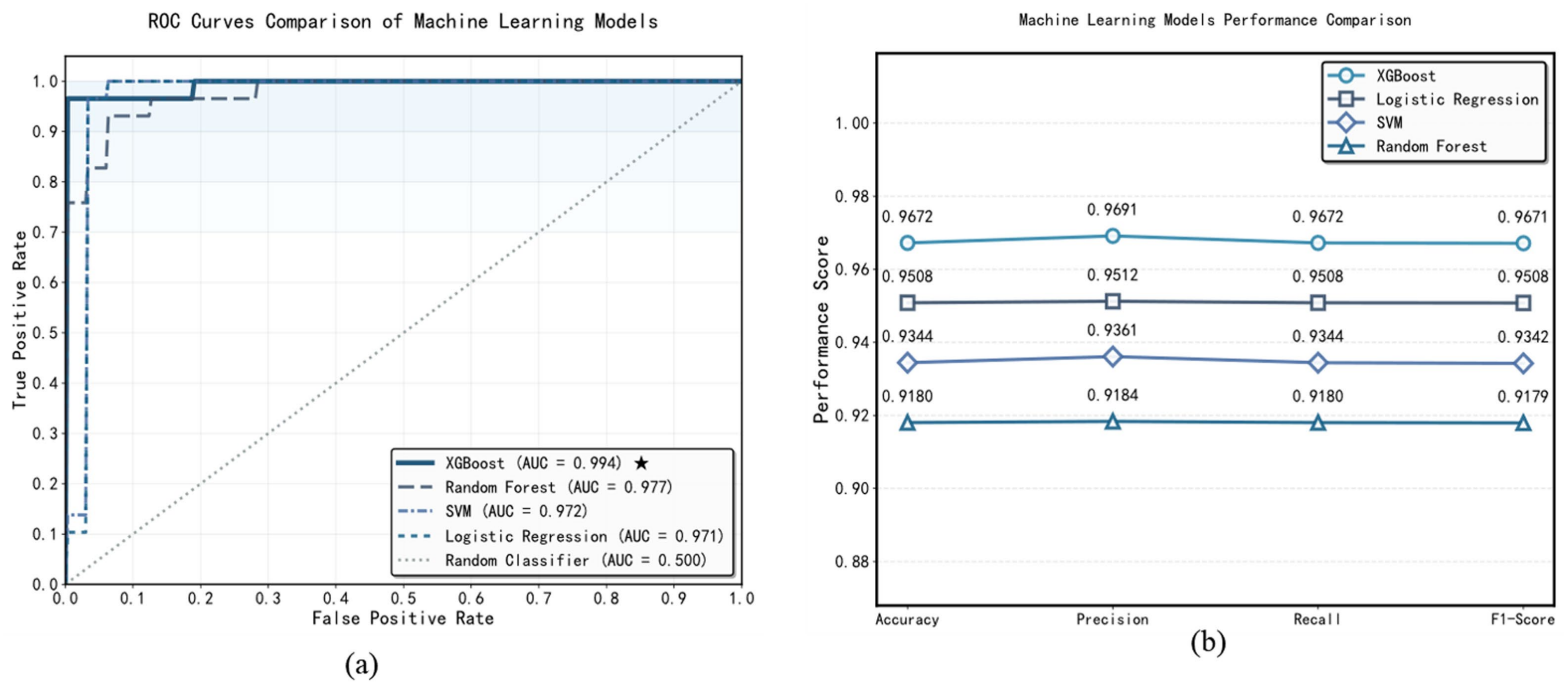
Comprehensive performance comparison of diagnostic models. **(a)** Comparison of ROC curves and area under the curve (AUC) for each model: ROC curves and AUC values provide a visual representation of each model’s ability to distinguish between subclinical depression and healthy controls. A higher AUC value indicates superior overall discriminative performance of the model. **(b)** Comparison of accuracy, precision, recall, and F1 score across models. The bar chart compares four commonly used evaluation metrics: accuracy (proportion of correctly classified cases), precision (accuracy of positive predictions), recall (detection rate of positive samples), and F1 score (harmonized average of precision and recall). All metrics are expressed as percentages (%), with error bars representing 95% confidence intervals. Results indicate that the Random Forest model achieves the highest AUC and F1 score, demonstrating optimal diagnostic performance on the current dataset.

The comprehensive evaluation metrics (Figure 4) further validate the superiority of XGBoost. Its area under the ROC curve (AUC) reached 0.994, significantly surpassing Random Forest (0.977), Support Vector Machine (0.972), and Logistic Regression (0.971). In ten-fold cross-validation based on the optimal subset of six EEG functional connectivity features (*β*-band DAN, *β*-band SMN-DAN, *α*-band SN, *β*-band SN-DMN, *α*-band SMN-DAN, *β*-band SMN-SN), XGBoost achieved an accuracy of 0.9672, with an F1 score of 0.9671 and precision of 0.9691, and recall of 0.9672. This ranks first among the four models. These results indicate that XGBoost effectively captures higher-order nonlinear interactive features among the attention network (DAN), salience network (SN), sensorimotor network (SMN), and default mode network (DMN) in adolescents with depressive symptoms through its gradient boosting framework. This achieves an optimal balance between predictive accuracy and clinical sensitivity, significantly reducing the risk of both missed and misdiagnosed cases.

In summary, the XGBoost model demonstrates significant advantages in terms of comprehensive classification performance, robustness, and interpretability. It has been identified as the optimal diagnostic model for this study, providing an efficient and reliable machine learning tool for early objective screening of depressive symptoms based on resting-state functional connectivity in electroencephalograms. This approach holds considerable potential for clinical translation.

#### 3.3.2 Intervention model

To predict the response of patients with depressive symptoms to exercise interventions based on their positive affect index, the predictive model employs six key indicators selected from 45 functional connectivity features through a three-stage feature selection process, as previously used in the diagnostic model (*β*-band DAN, *β*-band SMN-DAN, *α*-band SN, *β*-band SN-DMN, *α*-band SMN-DAN, *β*-band SMN-SN), supplemented by the baseline PA index as an additional affective feature, forming a seven-feature input set. As with the diagnostic model, four machine learning algorithms were employed: Support Vector Machine (SVM), Random Forest (RF), Logistic Regression (LR), and XGBoost. The distinction lies in the prediction model being preset as a three-class classification model. However, as the intervention results indicated zero participants in the low-response group, this study retained a binary classification model consistent with the diagnostic model. Model training employed an 8:2 split for the training and testing datasets. The sample labels were assigned as 0 for the moderate-response group and 1 for the high-response group.

**Figure 5:**
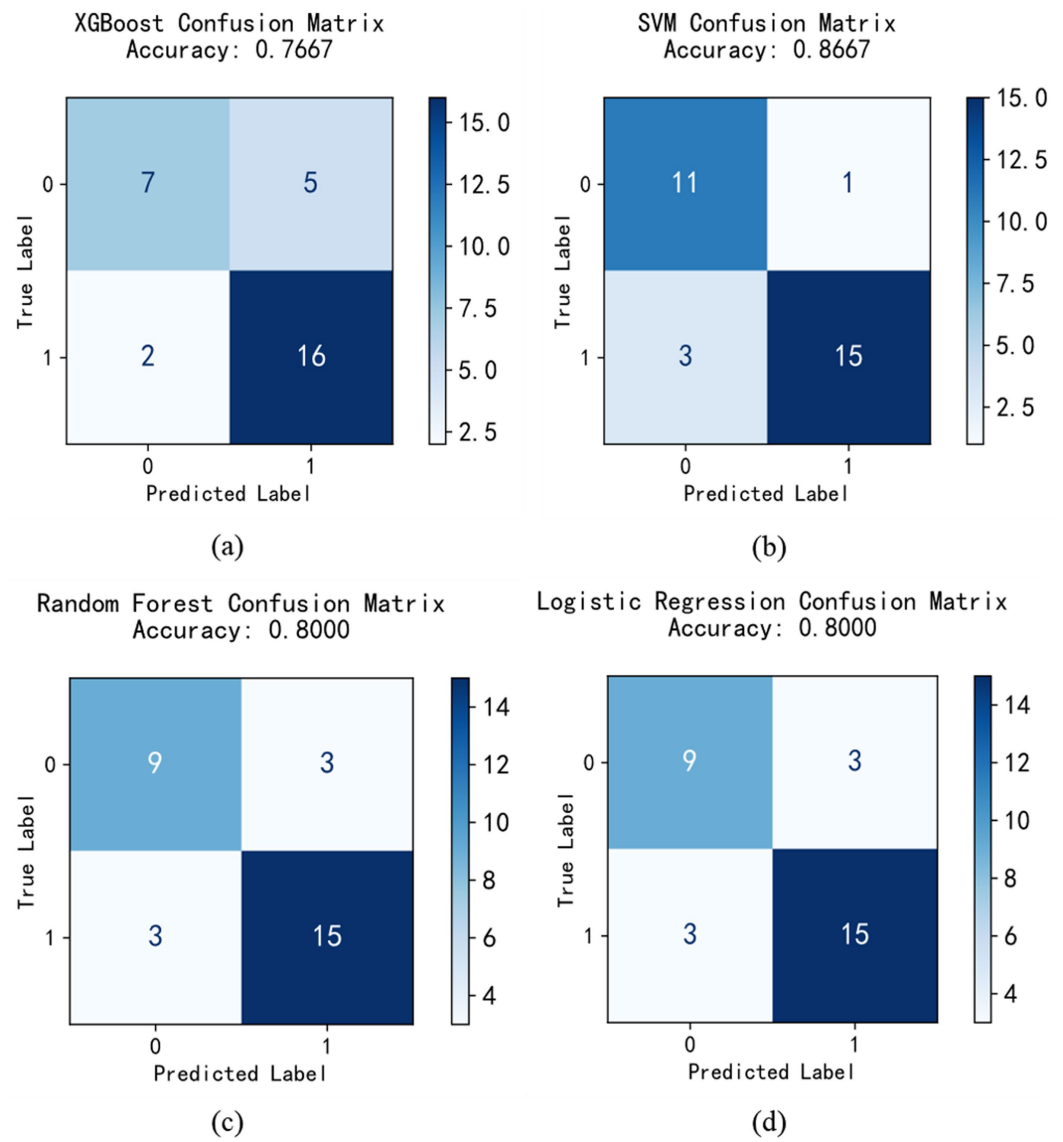
Confusion matrices of different machine learning models for intervention-response prediction. This figure presents the confusion matrices of the four predictive models on the test set: **(a)** XGBoost; **(b)** Support Vector Machine; **(c)** Random Forest; and **(d)** Logistic Regression. The horizontal axis represents the predicted class, and the vertical axis represents the true class. Class 0 indicates the moderate-response group, and class 1 indicates the high-response group. Diagonal values represent correctly classified samples, whereas off-diagonal values represent misclassified samples.

**Figure 6:**
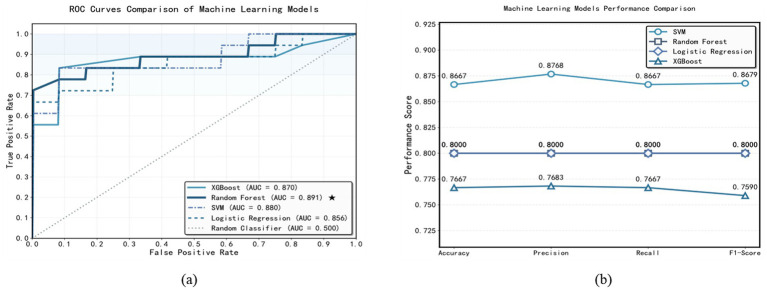
Performance comparison of machine learning models for intervention-response prediction. **(a)** ROC curves and AUC values of the four predictive models. Random forest achieved the highest AUC, indicating strong overall discriminative performance. **(b)** Comparison of accuracy, precision, recall, and F1-score across models. Although random forest showed the highest AUC, SVM achieved the best balance across clinically relevant metrics, particularly F1-score and recall. Therefore, SVM was selected as the preferred predictive model for distinguishing high responders from moderate responders.

Figure 5’s confusion matrix demonstrates that the SVM exhibits superior performance in both the true positive rate (TP) and the true negative rate (TN), while maintaining the lowest false positive rate (FN). This indicates its heightened sensitivity in identifying genuine beneficiaries, thereby mitigating the risk of erroneous clinical interventions. Although XGBoost achieved the highest true positive rate, it also exhibited the highest false positive rate, potentially leading to misclassification of moderate responders as high responders and thereby increasing the risk of undertreatment. The confusion matrices for the RF and LR models demonstrated relatively balanced performance. Subsequently, analyzing the ROC curves depicted in Figure 6a, the AUC values for the four models were: Support Vector Machine = 0.880, Random Forest = 0.891, Logistic Regression = 0.856, XGBoost = 0.870. This indicates that the dataset demonstrates high predictive performance across various machine learning algorithms, with the Random Forest model achieving the largest area under the ROC curve. Figure 6b further indicates that the SVM achieved an F1 score of 0.8679, an accuracy of 0.8667, a precision of 0.8768, and a recall of 0.8667, all significantly surpassing the other three models. Therefore, considering model stability, interpretability, and clinical applicability, the Support Vector Machine not only demonstrates excellent discriminative capability but also performs reliably on key medical decision-making metrics. It is thus the most recommended predictive model for this study.

### 3.4 Visual explanations for diagnostic and predictive models

#### 3.4.1 Diagnostic model

**Figure 7:**
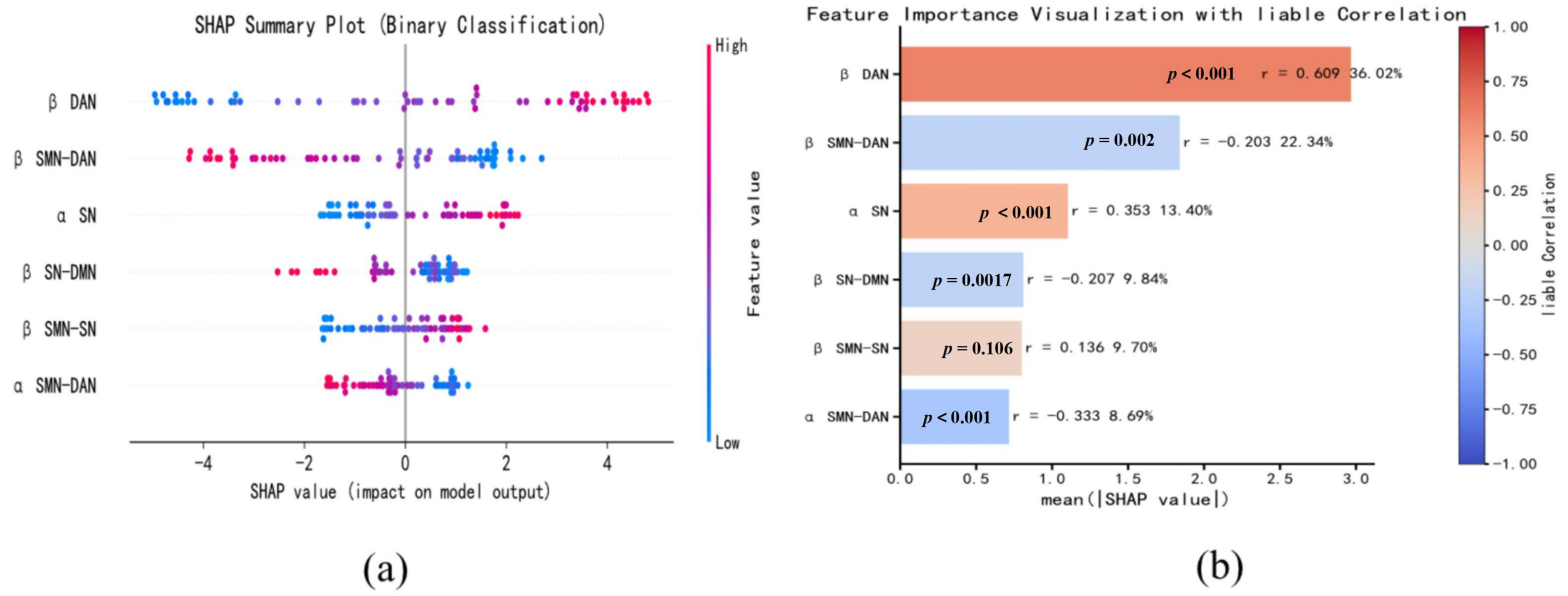
Global feature importance analysis and interpretation of the diagnostic model. **(a)** Feature summary plot based on SHAP values, **(b)** Feature importance ranking plot incorporating correlation indices. This figure presents the global feature importance analysis results for the depression diagnosis model. **(a)** The SHAP summary plot ranks features by importance from highest to lowest. The horizontal axis represents SHAP values (average contribution to model prediction), with each point denoting a sample. Color indicates feature value magnitude (red for high, blue for low), visually illustrating each feature’s overall contribution and directionality to model output. **(b)** Feature importance ranking diagram incorporating correlation indices. The vertical axis displays feature names, while the horizontal axis shows the mean absolute value of SHAP (mean |SHAP|). The depth of bar colors reflects the correlation indices between features (darker shades indicate higher correlation), aiding in the identification of potentially redundant features.

Analysis of the six key EEG features within the depression diagnostic model, using the SHAP framework (Figure 7), Combined with Figure 7a, the features are ranked by importance from highest to lowest. The horizontal axis represents the SHAP values (average contribution to the model’s prediction), and each point represents a sample. Colors indicate the absolute magnitude of the feature values (red indicates high, blue indicates low), as well as the feature importance ranking plot for the correlation metrics shown in Figure 7b. The vertical axis displays feature names, and the horizontal axis displays the average absolute SHAP values. The shade of the bar colors reflects the correlation between features indicates that the dorsal attention network (DAN) in the beta band dominates depression risk prediction (contribution: 36.02%). The feature importance is ranked as follows: *β*-band DAN (36.02%), *β*-band SMN-DAN (22.34%), *α*-band SN (13.40%), *β*-band SN-DMN (9.84%), *β*-band SMN-SN (9.70%), and *α*-band SMN-DAN (8.69%).

Further analysis using Bonferroni correction for multiple comparisons is shown in Figure 7b that *β*-band SMN-DAN (*r* = −0.203, *p* = 0.0022), *β*-band SN-DMN (*r*= −0.207, *p* = 0.0017), and *α*-band SMN-DAN (*r* = −0.333, *p* < 0.001) exhibited significant negative correlations, indicating that lower connectivity strength increases the model’s predictive probability for depression; whereas the *β*-band SMN-SN (*r* = 0.136, *p* = 0.106) did not reach statistical significance after adjustment. The *β*-band DAN (*r* = 0.609, *p* < 0.001) and the *α*-band SN (*r* = 0.353, *p* < 0.001) showed significant positive correlations, with reduced characteristic values increasing the risk of depression diagnosis.

**Figure 8:**
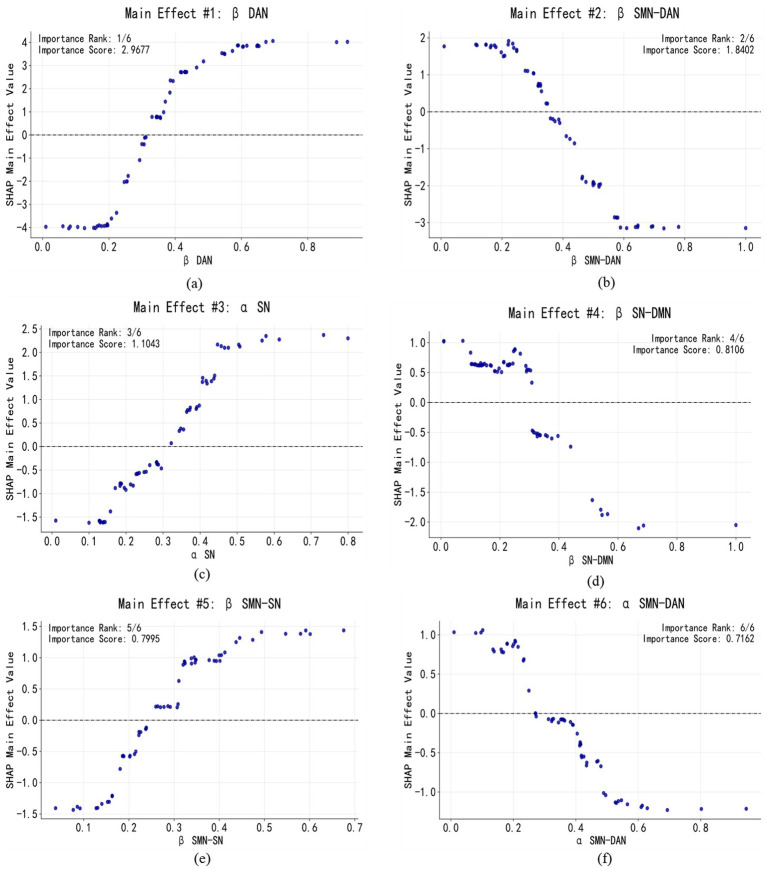
Main effects plot of key biomarkers in the diagnostic model. This figure presents the dependence plots for six key biomarkers selected in the depression diagnostic model: **(a)** β DAN; **(b)** β SMN-DAN; **(c)** α SN; **(d)** β SN-DMN; **(e)** β SMN-SN; and **(f)** α SMN-DAN. Each subplot displays the actual value of the feature on the x-axis, with the y-axis representing the SHAP value, indicating the feature’s contribution to the model’s prediction. Positive values drive positive predictions, whereas negative values drive negative predictions. The color of each scatter point denotes the partner feature that exhibits the strongest interaction with that variable, with purple to yellow indicating low to high interaction strength.

The SHAP main effects plot (Figure 8) reveals the non-linear relationship between key brain network features and prediction outcomes within the model. The *β*-band DAN, as the primary feature (importance score 2.9677), exhibits an overall S-shaped contribution curve. Its SHAP value increases significantly within the connection strength range of 0.2–0.6 and turns positive when the connection strength exceeds 0.3. This indicates that the *β*-band DAN connections of medium strength or above exert a pronounced positive driving effect on positive predictions, while contributions tend to stabilize at extremely low or high connection strengths.

Conversely, the beta band SMN-DAN (importance score 1.8402) exhibits a decreasing trend within the 0.2–0.6 range, suggesting that stronger SMN-DAN connectivity may suppress the model’s positive prediction bias. The alpha band SN (importance score 1.1043) exhibited an increasing trend within the 0.1–0.5 range, turning positive when connection strength exceeded 0.3. This indicates that alpha band SN connections of medium or greater strength contribute to enhancing the probability of positive predictions. The beta-band SN-DMN (importance score 0.8106) consistently exhibits a negative contribution (approximately −0.5 to 0) when connection strength exceeds 0.3, suggesting that stronger SN-DMN coupling may inhibit positive predictions.

The *β*-band SMN-SN connection (importance score 0.7995) exhibits predominantly negative SHAP values at low values (<0.3), indicating a tendency towards negative predictions by the model. At high values (>0.3), the values become positive and increase incrementally within the 0.1–0.4 range, suggesting that this connection becomes a key driver for positive predictions once it exceeds a certain threshold. Finally, the *α*-band SMN-DAN (importance score 0.7162) exhibits an inverted S-shaped profile, with SHAP values decreasing within the 0.2–0.6 range and approaching positive values when the connection strength falls below 0.25. This indicates that lower-intensity *α*-band SMN-DAN connections may enhance positive predictive tendencies. Collectively, these nonlinear relationships elucidate the differential contributions of brain network characteristics across distinct frequency bands and intensity ranges towards predicting depression, providing a refined interpretative framework for understanding the complex dynamic patterns of multi-frequency brain network abnormalities.

**Figure 9:**
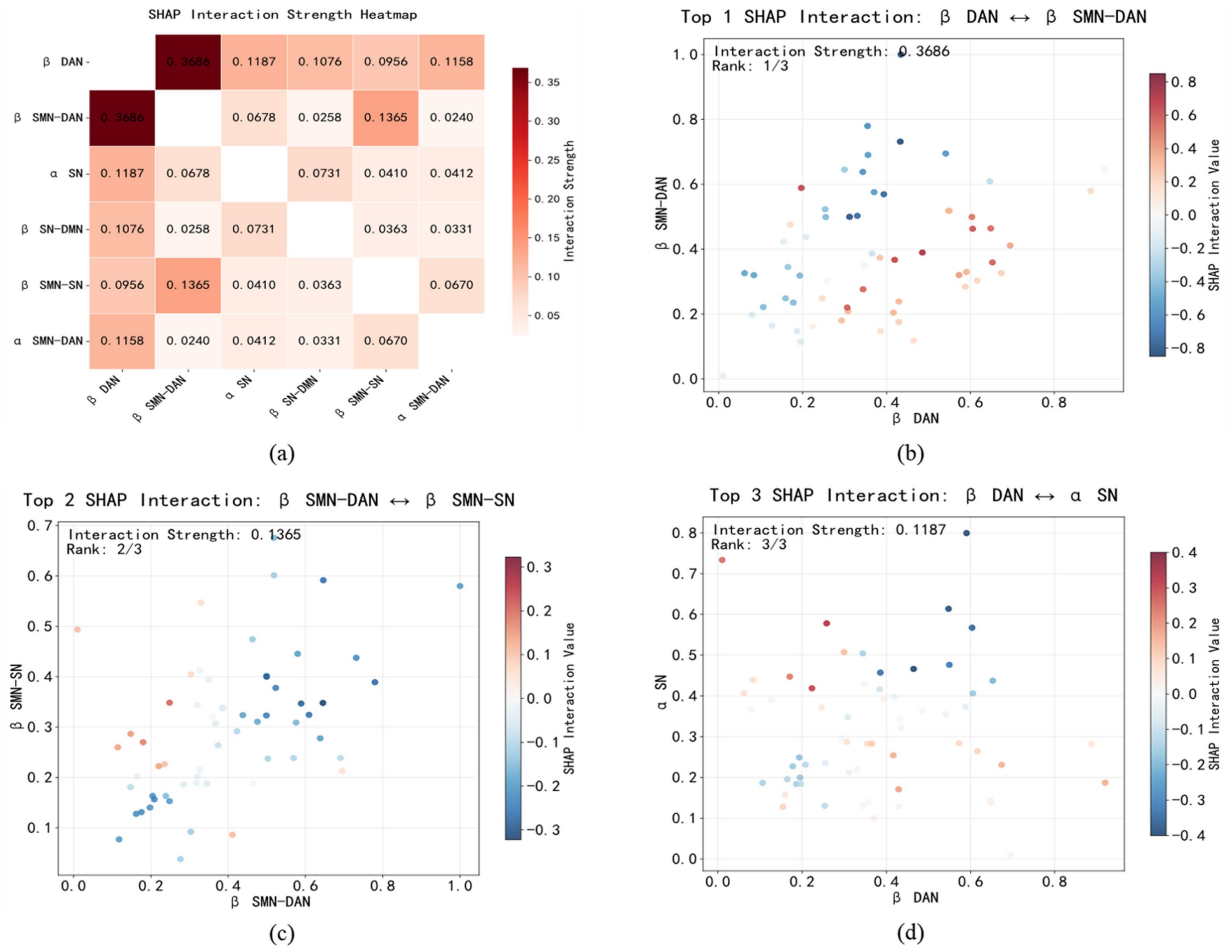
Interaction analysis of key biomarkers in the diagnostic model. This figure presents the results of interaction analysis for six key biomarkers within a depression diagnostic model. **(a)** Interaction strength heatmap, where the horizontal and vertical axes represent the six features, respectively. Cell color intensity indicates interaction strength (SHAP interaction value, with red denoting positive interaction and blue denoting negative interaction). Numerical annotations for interaction strength (IS) visually reflect synergistic or antagonistic effects between feature pairs. **(b–d)** Correspond to interaction dependence plots for the top three feature pairs by interaction strength. The horizontal axis displays a feature’s value, while the vertical axis shows its SHAP value. Scatter plot colors indicate the partner feature with the strongest interaction (purple → yellow denotes low → high), clearly revealing non-linear interaction patterns between features and their combined contribution to model prediction.

The SHAP interaction analysis further elucidated the patterns of synergy among key brain network features in the diagnostic model for depressive symptoms. The interaction strength heatmap (Figure 9a) shows that the strongest interaction occurs between the *β*-band DAN and the *β*-band SMN-DAN (IS = 0.3686), indicating that the dyssynchrony between the dorsal attention network and the sensorimotor attention network in the *β*-band is a core driving factor in the model’s predictions. Interactions of moderate strength were primarily observed between *β*-band SMN-DAN and *β*-band SMN-SN (IS = 0.1365), as well as between *β*-band DAN and *α*-band SN (IS = 0.1187), suggesting the presence of cross-frequency network modulation mechanisms. The interaction strengths for the remaining feature pairs were weaker (0.0331–0.0731), indicating that their contributions are relatively independent.

The interaction map further illustrates the synergy patterns of key feature pairs (Figure 9b): When the connection strength of *β*-band DAN falls within the range of 0.3–0.6, and the connection strength of *β*-band SMN-DAN falls within the range of 0.2–0.4, both exhibit significant positive interaction effects (SHAP value >0.2), as shown in Figure 9c, jointly enhancing the model’s contribution to positive predictions. Conversely, when the connection strength of the *β*-band SMN-DAN falls within the range of 0.4–0.8, and the connection strength of the *β*-band SMN-SN falls within the range of 0.25–0.45, the two exhibit a negative interaction, as shown in Figure 9d. Interactions among *β*-band DANs.

**Figure 10:**
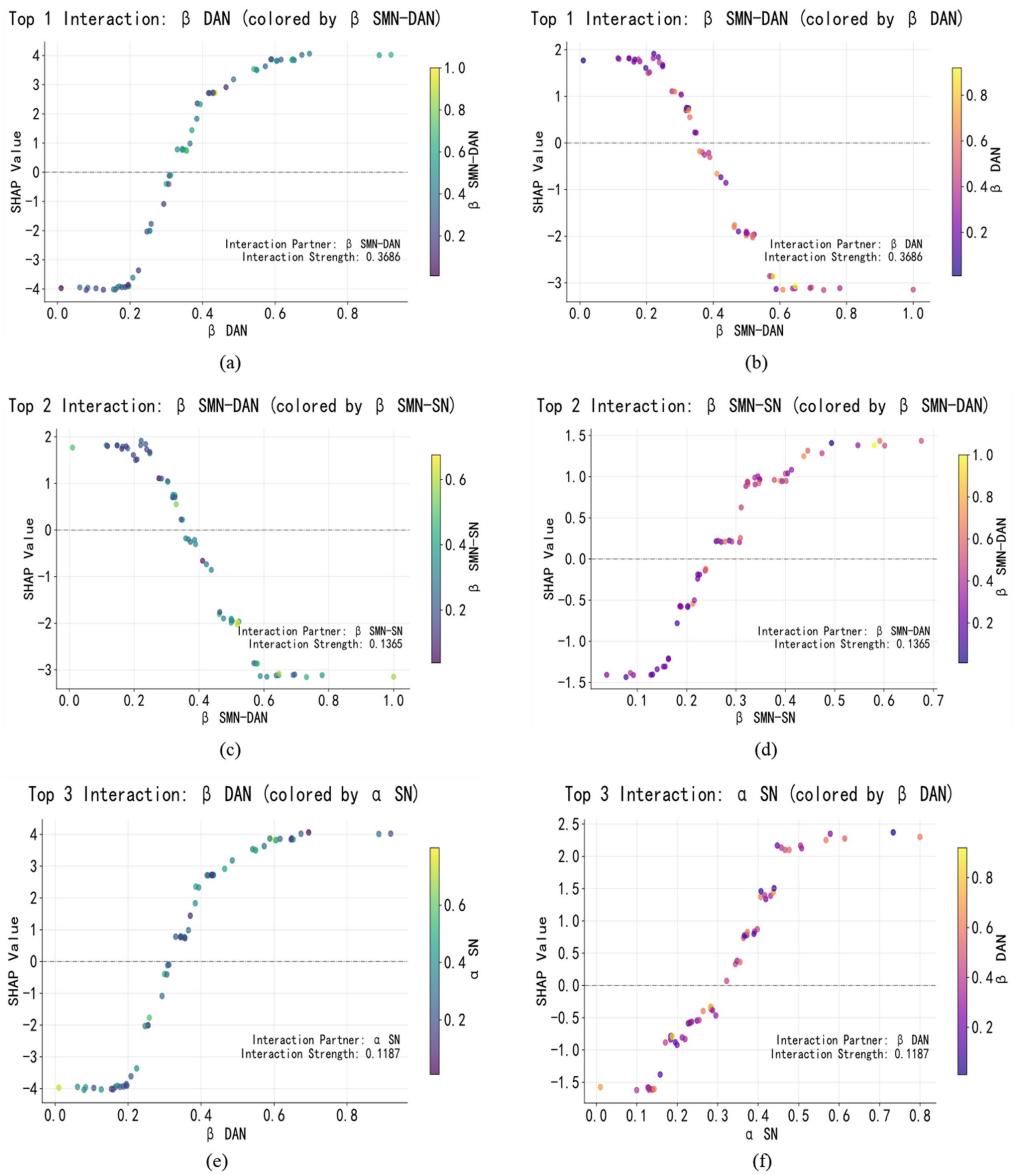
Diagnostic model interaction feature dependency diagram. **(a–f)** Interaction dependence plots for the top six feature pairs ranked by interaction strength. This figure presents interaction dependence plots for the top six key feature pairs ranked by interaction strength within the diagnostic model for subclinical depression. **(a)** *β*-Band DAN with *β*-band SMN-DAN; **(b)** *β*-band SMN-DAN with *β*-band SMN-SN; **(c)** *β*-band DAN with *α*-band SN; **(d)** *β*-band SN-DMN; **(e)** *α*-band SMN-DAN; **(f)** *β*-band SMN-SN. Each subplot displays the value of one feature on the *x*-axis and its SHAP value on the *y*-axis (positive values indicate promotion of positive predictions, negative values indicate promotion of negative predictions). The color of each scatter point corresponds to the partner feature exhibiting the strongest interaction with it (purple → yellow denotes low → high interaction strength.

The SHAP interaction dependency plots (Figures 10a–f) systematically reveal the nonlinear contributions of multi-frequency brain network features within the diagnostic model and their intricate interactive mechanisms, highlighting the synergistic, inhibitory, and dynamic gating effects between cross-frequency brain networks. The *β*-band DAN and *θ*-band SMN-DAN jointly form the core driving axis (interaction strength 0.3868). The *β*-band DAN eigenvalues exhibit a steep rise within the 0–0.4 range before plateauing towards saturation, demonstrating a typical nonlinear saturation effect; conversely, the *θ*-band SMN-DAN shows a gradual positive enhancement. Scatter points are colored by interaction partner (purple → yellow corresponding to low → high eigenvalues), with colors monotonically increasing along the x-axis and exhibiting high covariation, strongly validating the classical fork-collider cooperative structure (Figures 10a,b).

The *β*-band SMN-DAN, acting as a secondary unit (interaction strength 0.1365), forms a chain-forked extension via relaying through the *θ*-band SMN-DAN, exhibiting positive covariation in the scatter plot color (Figure 10d). Despite its relatively minor overall contribution, the *β*-band SMN-DAN exerts a crucial modulatory role: the *β*-band SMN-SN exhibits weak negative dependence with moderate color covariation, forming chain-like conduction pathways inhibited by the *β*-band SMN-DAN (Figure 10c). The *α*-band SN itself exhibits a stable positive contribution (interaction strength 0.1187), yet its color shows significant negative covariation, indicating it forms a cross-frequency inhibitory pathway by blocking the *β*-band DAN (Figure 10f). The *β*-band-DMN interaction (strength 0.1610) exhibits strong hierarchical properties, with its inhibition-coordination switching entirely driven by the *α*-band SMN-DAN, constituting a typical collider-like dynamic gating patterns (Figure 10e).

In summary, the multi-band brain network features of this diagnostic model do not constitute a simple linear superposition. Instead, they form a highly non-linear, hierarchical, and dynamically regulated interactive network. This network is driven by the core axis of the *β*-band DAN and the *θ*-band SMN-DAN, modulated by the *β*-band SMN-DAN, cross-frequency inhibition from the *α*-band SN, and dynamically gated by the *α*-band SMN-DAN and the *β*-band DMN. This intricate mechanisms provides crucial theoretical underpinnings and intuitive visual evidence for understanding the fine-tuned regulatory processes of brain networks in disease diagnosis.

#### 3.4.2 Predictive models

**Figure 11:**
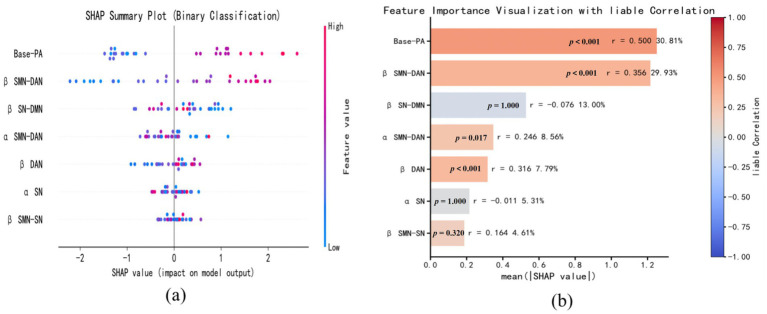
Global feature importance analysis and interpretation of the predictive model. **(a)** Feature summary plot based on SHAP values and **(b)** feature importance ranking plot incorporating correlation indices.

We evaluated the contribution and correlation of seven key features (Base-PA, *β*-band SMN-DAN, *β*-band SN-DMN, *α*-band SMN-DAN, *β*-band DAN, *α*-band SN, *β*-band SMN-SN) in the predictive model for depressive symptoms following a motor intervention, based on the SHAP (SHapley Additive exPlanations) framework. These features, quantified using phase-locked values (PLV), reflect alterations in brain network functional connectivity and emotional states before and after the exercise intervention. As shown in Figure 11, Combined with Figure 11a, the features are ranked by importance from highest to lowest. The horizontal axis represents the SHAP values (average contribution to the model’s prediction), and each point represents a sample. Colors indicate the absolute magnitude of the feature values (red indicates high, blue indicates low), as well as the feature importance ranking plot for the correlation metrics shown in Figure 11b. The vertical axis displays feature names, and the horizontal axis displays the average absolute SHAP values. The shade of the bar colors reflects the correlation between features. Base-PA dominates the prediction model (contribution: 30.81%), while the brain network feature *β*-band SMN-DAN (contribution: 29.93%) plays a secondary role.

The remaining features ranked in order of importance were: the *β*-band SN-DMN (13.0%), the *α*-band SMN-DAN (8.56%), the *β*-band DAN (7.79%), the *α*-band SN (5.13%), and the *β*-band SMN-SN (4.61%). Further quantitative analysis of the correlations between pre-intervention brain networks, clinical manifestations, and treatment efficacy revealed that Base-PA (*r* = 0.500, *p* < 0.001), *β*-band SMN-DAN (*r* = 0.356, *p* < 0.001), *α*-band SMN-DAN (*r* = 0.246, *p* = 0.017), and *β*-band DAN (*r* = 0.316, *p* < 0.001) were significantly positively correlated with intervention efficacy, while *β*-band SMN-SN (*r* = 0.164, *p* = 0.320) showed a positive correlation but did not reach statistical significance; This suggests that, within a certain range, higher pre-intervention scores are associated with better intervention outcomes. Conversely, the *β*-band SN-DMN (*r* = −0.076, corrected *p* = 1.000) and the *α*-band SN (*r* = −0.011, *p* = 1.000) showed negative correlations, indicating that, within a certain range, lower pre-intervention scores lead to more favorable intervention outcomes. All correlations mentioned above were corrected using the Bonferroni multiple comparison correction, and corrected *p*-values are reported to ensure statistical rigor.

**Figure 12:**
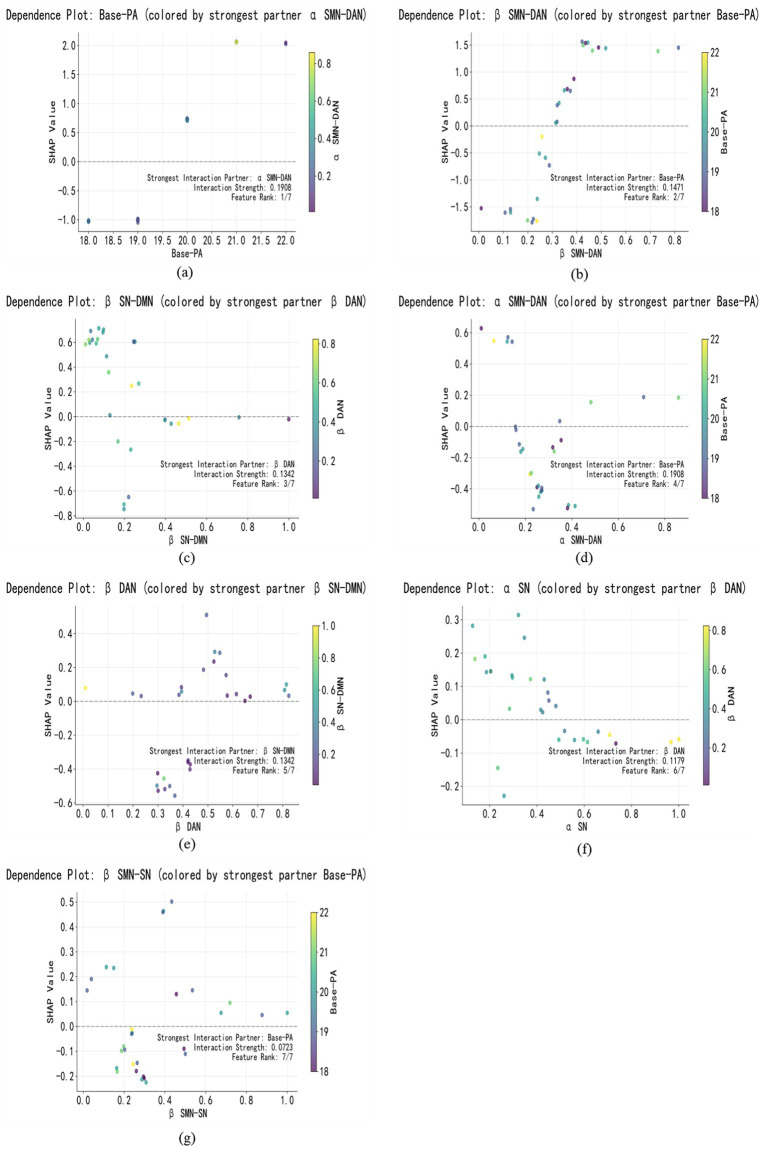
Interaction feature dependency diagram of the predictive model. This figure presents dependence plots for the seven key predictive factors identified in the exercise intervention efficacy prediction model: **(a)** Base-PA; **(b)** β SMN-DAN; **(c)** β SN-DMN; **(d)** α SMN-DAN; **(e)** β DAN; **(f)** α SN; and **(g)** β SMN-SN. Each subplot displays the actual value of the feature on the x-axis and the SHAP value on the y-axis. Positive values indicate a prediction of high response, whereas negative values indicate a prediction of low response. The color of each data point corresponds to the partner feature exhibiting the strongest interaction with it, with purple to yellow indicating low to high interaction strength. The subplots reveal the nonlinear relationships and interaction patterns between features and model outputs, providing an intuitive and interpretable perspective for understanding the contributions and synergistic patterns of baseline positive affect levels and multi-band brain network features in predicting intervention efficacy.

The SHAP dependency plots (Figures 12a–g) systematically reveal the nonlinear contributions of seven key features within the predictive model for exercise intervention outcomes, alongside their interactive mechanisms. Baseline physical activity (PA) level not only contributed significantly independently, but also drove inhibitory integration through a strong interaction with the *α*-band SMN-DAN (strength 0.1908) (negative color-coded covariation, purple → yellow with a monotonic increase along the horizontal axis). It further up-regulated the *β*-band SMN-DAN via chain enhancement (strength 0.1471) (non-linear positive, peak +1.4, positive color covariation), forming a cross-frequency closed loop between PA↔*α*/*β* SMN-DAN (total strength 0.3379). This establishes the core foundation for behavioral-neural prediction (Figures 12a,b,d). The *β*-band DAN-SN-DMN constitutes the core neural axis (bidirectional interaction 0.1342): DAN’s predictive value is highly dependent on SN-DMN synergy (strong positive color covariation), with the latter’s weak positive effect entirely mediated by DAN. This achieves dynamic gating between the attention and default mode networks via a ‘V’-shaped inversion, governing intervention sensitivity (Figures 12c,e). The predictive role of the *β*-band SMN-SN interaction is relatively marginal (interaction strength 0.0723), with its value primarily manifested through SMN-DAN relaying (Figure 12f). The SN main effect in the *α*-band approaches zero, primarily providing negative covariate auxiliary modulation via the *β*-band DAN inhibitory chain (strength 0.1179) (Figure 12g).

Integrating Figure 11, the predictive model for exercise intervention effects employs the PA-*α*/*β* SMN-DAN cross-frequency hub as the behavioral entry point, with the *β* DAN-SN-DMN bidirectional closed loop serving as the core neural axis. This is supplemented by SMN internal expansion and *α*-band distributed inhibition, collectively constructing a complete causal map: “behavioral drive → core coordination → dynamic gating → frequency band equilibrium”. This architecture aligns closely with the d-separation and path activation principles within probabilistic graphical models, providing a systematic, interpretable, multi-scale theoretical and empirical framework for personalized motor intervention design, elucidating neuroplasticity mechanisms, and enabling precise depression prediction.

## 4 Discussion

### 4.1 Discussion of results from screening the optimal feature subset for diagnostic models

The patterns identified in this study—including the central contribution of the *β*-DAN, the cross-frequency inhibition of the *β*-DAN by the *α*-SN, and the dynamic gating of the *α*-SMN-DAN—must be interpreted within the unique context of neurodevelopment during adolescence. Adolescence is a peak period of neural plasticity, characterized by the ongoing maturation of the prefrontal cortex, significant reorganization of the dorsal attention network (DAN) and the salience network (SN), and dynamic changes in theta-alpha oscillations. These processes enhance network variability and vulnerability. Subclinical depression may amplify multi-frequency, multi-network dyssynchrony within these developmental windows of sensitivity, leading to early deficits in emotional regulation. The findings of this study not only reveal the early neurophysiological basis of these mechanisms but also provide EEG evidence for developmental neuroscience: motor interventions may promote prefrontal-sensorimotor integration and enhance plasticity recovery by modulating *β*-SMN-DAN connectivity. This discovery broadens clinical relevance and supports targeted interventions in early adolescence to interrupt depressive trajectories.

This study is essentially an exploratory study that employs a fully data-driven machine learning framework to perform *a priori*-free screening of functional connectivity across five networks in resting-state EEG. Although we proposed explicit neurobiological hypotheses in the introduction based on the existing literature, the final findings are primarily data-driven. Therefore, the patterns discussed in the Discussion section, such as the “fork-collision synergy structure” and “cross-frequency inhibition,” should be regarded as model-based exploratory insights and testable hypotheses, rather than confirmatory evidence of known mechanisms. This adjustment allows this work to achieve a better integration between data-driven methods and neurobiological theoretical foundations. This study aims to explore the neurophysiological mechanisms underlying depressive symptoms and develop an objective diagnostic model based on resting-state functional connectivity in the brain. By analyzing both intra-network and inter-network connectivity within five core brain networks (SMN, DAN, SN, DMN, CEN) across the theta, alpha, and beta frequency bands, we employed a multi-stage feature selection strategy. This ultimately yielded an optimal biomarker combination comprising six key features. This combination effectively distinguishes individuals with depressive symptoms from healthy controls, offering new insights into the early pathophysiological changes of depression and enabling early intervention.

The optimal feature set identified by the study primarily concerns the beta and alpha frequency bands, highlighting the central role of the dorsal attention network (DAN), salience network (SN), and sensorimotor network (SMN) in depressive symptoms. This finding not only partially aligns with previous research outcomes on major depressive disorder (MDD) (Dosenbach et al., 2025). It also reveals unique patterns of neural circuit abnormalities that may exist during the subclinical stage. Firstly, enhanced functional connectivity within the beta-band dorsal anterior cingulate network (DAN) represents the most significant biomarker identified in this study. The DAN primarily governs top-down attentional allocation and cognitive control. In depressed individuals, the difficulty in shifting attention away from negative information constitutes one of their core cognitive deficits (Xu et al., 2024). The *β*-band neural oscillations are typically associated with active cognitive processing, motor readiness, and cortical excitability states (Nusslock et al., 2024). Therefore, the abnormal enhancement of beta band connectivity within the DAN may reflect that individuals with subclinical depression require greater neural effort to sustain attention or perform cognitive tasks, or exhibit a state of cognitive rigidity and hypervigilance. This correlates with the ruminative thinking and attentional bias commonly observed in depression.

Secondly, connectivity features involving the SN, such as intra-SN connections within the alpha band and SN-DMN connections in the beta band, also demonstrate considerable significance. The SN functions as a “switching pattern” in identifying and integrating salient stimuli from both internal and external sources, thereby guiding subsequent allocation of cognitive resources. Depression is frequently conceptualized as a “salience disorder,” wherein individuals tend to assign disproportionately high salience to negative emotional information (Li et al., 2024). Alpha band oscillations are closely associated with neural inhibitory functions and the filtering of irrelevant information. Consequently, alterations in alpha band SN internal connectivity may indicate dysfunction in regulating information influx and suppressing extraneous interference. Concurrently, the dynamic equilibrium between the SN and DMN is crucial for flexible switching between internally directed self-reflection (DMN-dominant) and externally oriented task processing (CEN/DAN-dominant). Previous studies have repeatedly demonstrated that abnormal SN-DMN connectivity in patients with major depressive disorder correlates with excessive rumination (Wei et al., 2024). This study identified alterations in beta-band SN-DMN connectivity during the subclinical stage, further suggesting that such network dysregulation may emerge in the early stages of the disease.

Finally, this study also identified multiple SMN-related connectivity features, such as *β*-band SMN-DAN, *α*-band SMN-DAN, and *β*-band SMN-SN connections. The SMN primarily processes sensory inputs and motor outputs. In depression, somatic symptoms (such as fatigue and pain) alongside psychomotor retardation or agitation are common clinical manifestations (Singh et al., 2023). Abnormalities in SMN connectivity with the DAN and SN may reflect disturbances in the processing of somatosensory signals and their integration with attentional and salience assessment systems. For instance, alterations in SMN-SN connectivity could lead to normal interoceptive signals being erroneously flagged as “salient” or “threatening,” thereby amplifying perceptions of physical discomfort.

Abnormalities in SMN-DAN connectivity may be associated with psychomotor symptoms or difficulties in attention regulation during action execution. These findings provide neurocircuitry-level evidence for understanding the mind–body interaction pattern in depression. They both align with and diverge from existing literature on functional connectivity abnormalities in depression. Numerous studies have highlighted dysregulation within the ‘triple network model’ comprising the default mode network (DMN), central executive network (CEN), and salience network (SN) in major depressive disorder (MDD) (Castro et al., 2023). This study similarly identified the significance of connectivity between the SN and DMN, supporting the core tenets of this model. However, unlike previous research that focused more on DMN-intrinsic connectivity, our findings indicate that the DAN and its interactions with the SMN and SN play a more dominant role in identifying depressive symptoms. This may suggest that during the subclinical stages of the disorder, impairments in cognitive control and attentional modulation (DAN-related) alongside abnormalities in mind–body interactions (SMN-related) could represent more prominent or earlier-emerging neural markers. Research on depressive symptoms remains relatively scarce, yet existing evidence is beginning to reveal its distinct model-derived neurophysiological patterns. For instance, one study identified distinct patterns of altered thalamic-temporal functional connectivity between depressive symptoms and major depressive disorder (Grehl et al., 2023). Another study based on electroencephalogram successfully utilized connectivity features to classify depressive symptoms, emphasizing the importance of theta-band connectivity (Kelley et al., 2023). The findings of this study complement these discoveries by systematically revealing, for the first time across multiple frequency bands and network frameworks, the pivotal role of connectivity patterns centered on the beta band and DAN in depressive symptoms. This provides more refined evidence for constructing a neuropathological model of this disease stage.

### 4.2 Discussion of diagnostic models and predictive models

In this study, we successfully constructed and validated two machine learning models based on resting-state electroencephalogram (EEG) functional connectivity features, respectively for diagnosing depressive symptoms (SCD) and predicting response to exercise intervention. The results indicate that the XGBoost model demonstrated exceptional performance in distinguishing SCD patients from healthy controls (HC), achieving an AUC value of 0.994. Conversely, the Support Vector Machine (SVM) model yielded optimal accuracy and F1 scores when predicting SCD patients’ response to exercise intervention. These findings not only validate the feasibility of utilizing EEG functional connectivity as a neurobiological biomarker for identifying depressive symptoms, but also provide novel perspectives and tools for achieving precision-based, individualized mental health interventions.

One of the core contributions of this study is the identification of a set of EEG functional connectivity features capable of efficiently diagnosing SCD. These features primarily involve connectivity within the attention network (DAN), salience network (SN), sensorimotor network (SMN), and default mode network (DMN) across the alpha and beta frequency bands. Neuropathological research on depression widely recognizes that its core symptoms are closely associated with functional dysregulation across multiple large-scale brain networks. (Liu et al., 2025). For instance, excessive activity in the DMN is associated with rumination. The SN plays a pivotal role in emotional processing and the integration of internal and external environmental information, while the DAN is linked to cognitive control and attention deficits (Yang et al., 2025). This study found that during depressive episodes, subtle alterations in the connectivity patterns between these networks emerge, which can be captured by machine learning models. The superior performance of the XGBoost model in this diagnostic task suggests a complex nonlinear relationship between these brain network features and SCD status, which traditional linear models struggle to effectively characterize. This finding aligns with other studies employing machine learning and EEG functional connectivity to classify neuropsychiatric disorders (such as obstructive sleep apnea and attention deficit hyperactivity disorder), all demonstrating the powerful capability of nonlinear models in decoding intricate patterns of brain activity (Thorup et al., 2024).

Research focusing on depressive symptoms holds significant public health implications. Subclinical or subthreshold depressive states are recognized as major risk factors for developing major depressive disorder (MDD), often accompanied by functional impairment and diminished quality of life (Leung and Weiss, 2025). Among adolescents, where emotional fluctuations are commonplace, subclinical symptoms are more readily overlooked, thereby missing the optimal window for intervention (Liang et al., 2023). Currently, the identification of sudden cardiac death (SCD) relies primarily on subjective scales, lacking objective biological markers. The diagnostic model developed in this study utilizes portable, non-invasive, and relatively low-cost electroencephalogram (EEG) technology, offering the potential for objective screening of SCD. With an accuracy rate of up to 96.7% and an AUC value of 0.994, this model demonstrates potential as a clinical diagnostic aid. It facilitates the identification of high-risk individuals during the early, atypical symptom phase, enabling proactive interventions that may prevent or delay the onset of major depressive disorder (MDD). This aligns with prior research objectives using EEG signals to detect subclinical depression, while achieving superior classification performance (Gruskin et al., 2020). Furthermore, research indicates that cognitive deficits in patients with major depressive disorder may predate the onset of the illness or correlate with the severity of subclinical symptoms (Danneel et al., 2020). Therefore, early identification based on neurophysiological indicators is all the more crucial.

Another innovation of this study lies in the development of a predictive model for exercise intervention responses. Precision medicine represents the direction of modern medical advancement, and forecasting the efficacy of specific treatments is crucial for achieving personalized care. Our predictive model integrates baseline EEG functional connectivity features with positive affect (PA) indices, successfully distinguishing moderate responders from high responders.

Interestingly, in predictive tasks, SVM outperforms XGBoost, which excels in diagnostic tasks. This may reflect differences in the model-derived neurophysiological patterns underlying diagnosis and prognostic prediction. Diagnostic models must identify classification boundaries across broad heterogeneity from non-disease to disease states, potentially involving more complex nonlinear patterns – precisely where ensemble learning models like XGBoost demonstrate their strengths. Prognosis prediction involves identifying specific patterns associated with intervention response within a relatively homogeneous patient cohort (all with SCD), where the feature space may be relatively simple. By seeking the maximum margin hyperplane, SVM achieves a more robust classifier with superior generaliza0tion capabilities for this task. This finding underscores the necessity for systematic comparison across different clinical problems to select the most suitable machine learning algorithm based on data characteristics and task objectives, rather than blindly adhering to a single “optimal” model (Elbakary et al., 2025; Kim and Lee, 2025). Although the XGBoost diagnostic model achieved exceptionally high performance (AUC = 0.994, accuracy = 96.7% in 10-fold cross-validation on the optimal six-feature subset), we acknowledge a critical limitation raised by the observation that even the top two features alone (intra-*β*-DAN and *θ*-SMN-DAN connectivity) were sufficient to yield accuracies of approximately 93–95%. This level of classification performance approaches or exceeds the known test–retest reliability of resting-state EEG functional connectivity measures (typically ICC = 0.5–0.8 across theta/alpha/beta bands in healthy and clinical adolescent samples) and the moderate reliability of the self-report clinical instruments used to define group labels (CES-D and PHQ-9 test–retest correlations commonly range between 0.70–0.90). Such a discrepancy raises the possibility of model overfitting to sample-specific noise or spurious correlations rather than capturing robust, generalizable neurophysiological signals. To mitigate this risk, we implemented multiple safeguards throughout the analytical pipeline, including strict separation of an independent test set (20% hold-out, *N* = 61) prior to any feature selection or hyperparameter tuning, pipeline-based standardization within each cross-validation fold, and feature selection performed exclusively on the training set. Nevertheless, we recognize that these precautions do not fully eliminate the fundamental ceiling imposed by the reliability of input features and ground-truth labels. Future multi-center validation studies using larger, independent cohorts and incorporating explicit test–retest EEG datasets will be essential to establish the true upper bound of generalizability. In the interim, the biological plausibility and interpretability of the identified features—supported by SHAP interaction analyses revealing consistent multi-frequency, multi-network dyssynchrony patterns—provide preliminary reassurance that the model is capturing clinically meaningful variance rather than purely statistical artifacts. These limitations underscore the need for cautious interpretation when translating such high-accuracy models into clinical decision-support tools.

It is worth emphasizing that the interactive patterns revealed by SHAP in this study—including the “fork-collision synergy structure,” the cross-frequency inhibition of the *α*-SN on the *β*-DAN, and “dynamic gating”—are all based on the decision logic of XGBoost/SVM models and represent model interpretations at the level of correlation rather than rigorous causal inferences. Although these patterns are highly consistent with dysregulation in attention networks, salience networks, and sensorimotor networks reported in the existing literature, and provide clear testable hypotheses for future longitudinal, intervention, or causal modeling studies, their biological causality still requires further validation through methods such as transcranial magnetic stimulation, longitudinal tracking, or animal models.

### 4.3 Visual analysis and discussion of diagnostic and predictive models

This study successfully constructed and validated two machine learning models based on resting-state electroencephalogram (EEG) functional connectivity features: one for the auxiliary diagnosis of depression, and another for predicting the efficacy of exercise interventions on subclinical depression. By incorporating the SHAP framework, we not only achieved high-precision classification and prediction, but more significantly, unveiled the intricate model-derived neurophysiological patterns underpinning model decisions. This provides novel insights into the pathophysiology of depression and the neuroplasticity effects of exercise interventions.

Our research confirms that machine learning models can effectively distinguish between individuals with depression and healthy controls, consistent with previous studies utilizing electroencephalogram signals for automated identification of depression (Wang et al., 2025). However, the core contribution of this study lies in transcending the limitations of traditional “black-box” models by employing the SHAP framework to conduct an in-depth analysis of the model-derived neurophysiological patterns underlying model decision-making (Lei et al., 2025). The application of explainable artificial intelligence (XAI) methods enables us to quantify the contribution of each feature and visualize their complex interactions, which is becoming increasingly significant in neuroscience and clinical research.

The analysis results unequivocally indicate that the functional connectivity within the dorsal attention network (DAN) in the beta band constitutes the most significant predictive feature within the diagnostic model for depression. The DAN plays a pivotal role in cognitive control and goal-directed attentional allocation; its functional abnormalities are closely associated with core depressive symptoms - immersive rumination and attentional bias towards negative information (He et al., 2022). SHAP analysis indicates that moderate to high-intensity beta-band DAN connectivity significantly increases the probability of predicting depression, potentially reflecting compensatory hyperactivation within the brain to sustain cognitive function during depressive states (Yun and Kim, 2021). Or, be mindful of the decline in internal coordination efficiency within the network.

More intriguingly, the model reveals intricate nonlinear and interactive relationships between features. The robust interaction between the beta-band dorsal anterior network (*β*-DAN) and the sensorimotor network (*β*-DAN and *β*-SMN-DAN) emerges as the core driver of the model’s predictions. This dyssynergia between the attentional and sensorimotor networks may correspond to the psychomotor retardation or agitation symptoms commonly observed in depression (Wang et al., 2019). Moreover, the model identified cross-band regulatory mechanisms, such as the inhibitory influence of the alpha-band salience network (SN) on the beta-band default mode network (DAN). The alpha band is typically associated with neural inhibitory functions, while the SN governs attentional switching between internal and external sensory signals. This inhibitory pathway from the alpha-SN to the beta-DAN may indicate that, during depressive states, the equilibrium between the brain’s internal state monitoring network (SN) and its external task-processing network (DAN) becomes disrupted (Ellis et al., 2017). this has resulted in the inefficient allocation of cognitive resources.

The SHAP dependency graph further integrates these interactions into a highly structured dynamic network. At the model’s core lies a ‘fork-collider’ cooperative structure comprising the *β*-band DAN and *θ*-band SMN-DAN, dynamically gated and modulated by other networks such as the *α*-SN and *β*-DMN. This indicates that the neural basis of depression is not an isolated abnormality within a single network or frequency band, but rather a complex system of multi-frequency, multi-network dysregulation. This systemic, interpretable perspective provides a refined framework for understanding the pathophysiology of depression that transcends traditional linear models, and offers potential targets for future network-modulation-based neurointerventions. Beyond diagnostic models, this study successfully constructed a predictive model for the efficacy of exercise interventions in alleviating depressive symptoms. This exploration responds to the call for precision medicine, aiming to identify subgroups within the heterogeneous population of depression patients most likely to benefit from specific therapies.

Model results indicate that baseline physical activity levels (Base-PA) and beta-band SMN-DAN functional connectivity are the two most significant predictors of intervention outcomes. This underscores the equal importance of behavioral and neural indicators, supporting the bio-psycho-social medical model. Individuals with higher baseline PA levels demonstrated superior intervention responses, potentially reflecting their stronger behavioral activation foundation and motivational levels (Alavinikoo et al., 2025). The SMN-DAN connection in the beta band, as a core neural predictor, may represent the brain’s sensitivity and plasticity to motor inputs.

SHAP analysis further revealed a complete causal map of ‘behavioural drive → core coordination → dynamic gating → frequency band balance’. Baseline PA, through cross-frequency hubs with the *α*/*β*-band SMN-DAN, ‘injects’ behavioural information into the brain network, forming the behavioural input to the predictive model. This suggests that the effects of motor intervention may first manifest in the regulation of the sensorimotor system. Subsequently, the core neural axis formed by the *β*-band DAN and SN-DMN performs a crucial dynamic gating function, determining the brain’s sensitivity to this regulation. The antagonistic relationship between the DAN and DMN underpins healthy brain function, and disruption of this dynamic equilibrium is considered one of the core pathologies of depression (Wang et al., 2019). Our model indicates that individuals capable of effectively regulating the dynamic equilibrium of this core axis are more likely to benefit from exercise interventions.

This interpretable predictive model offers the potential for personalized exercise prescription. By assessing patients’ baseline PA levels and specific brain network characteristics, clinicians can make more informed judgments regarding whether exercise therapy constitutes the preferred intervention. Furthermore, tailored exercise programs—such as mindfulness-based coordination exercises—can be designed according to specific network dysregulation patterns (e.g., DAN-DMN gating failure). This approach paves the way for transitioning from one-size-fits-all treatment guidelines to data-driven, individualized interventions.

The primary strength of this study lies in its deep integration of advanced machine learning techniques with explainable AI frameworks. Unlike many studies solely focused on classification accuracy, we employed SHAP to translate complex model decision processes into intuitive, comprehensible neuroscientific hypotheses, providing clear guidance for subsequent mechanism research and clinical translation. Furthermore, we concurrently constructed diagnostic and intervention prediction models, demonstrating the method’s potential across the entire clinical application workflow.

## 5 Conclusion

In summary, this study successfully constructed an objective diagnostic model for subclinical depression (SCD) by utilizing resting-state functional connectivity features in electroencephalograms, combined with an explainable machine learning framework (SHAP). (XGBoost, AUC = 0.994) and a predictive model for exercise intervention efficacy (SVM). It also systematically reveals, for the first time, a multi-frequency, multi-network nonlinear interaction patterns centered on the dorsal attention network (DAN) in the *β* band, with the sensorimotor-dorsal attention network (SMN-DAN) in the *θ* band acting as a synergistic axis. This mechanism encompasses bifurcation-collider synergistic structures, cross-frequency inhibitory pathways, and dynamic gating patterns. These findings not only deepen our understanding of the early neuropathophysiology of depression but also validate the immense potential of EEG functional connectivity as a low-cost, non-invasive biomarker, providing a robust foundation for achieving early screening and personalized interventions.

This study transferred the optimal features from the diagnostic model to the predictive model, primarily based on the assumption of shared mechanisms and considerations of clinical utility; however, this strategy has limitations, and independent feature selection could be conducted in future studies to validate it. It is worth noting that the Schaefer-78 template used in this study was derived from adult data; although the average age of participants was 15.6 years, the adolescent brain is still developing, which may lead to slight deviations in brain region localization; Furthermore, despite the implementation of multiple anti-leakage strategies—including source space, sLORETA, and average reference—a small amount of residual volume-based conduction effects may still persist under low-density EEG conditions. Given the dynamic nature of adolescent brain network development, future studies could incorporate a longitudinal design and combine development-specific atlases, multimodal imaging, and debiased wPLI/iCoh metrics to further validate the age-related evolution of network patterns. Other limitations of this study include: small sample size, cross-sectional design limiting causal inference, limited spatial resolution of EEG, exclusion of low/non-responders from the predictive model, and the need for experimental validation of the biological causality inferred by SHAP. Looking ahead, this study provides a direction for precision mental health: conducting longitudinal cohort studies to validate the predictive value of EEG for depression conversion, constructing multimodal models, and developing a clinical decision support system that integrates diagnosis, risk assessment, and personalized intervention.

## Data Availability

The raw data supporting the conclusions of this article will be made available by the authors, without undue reservation.
